# Innovative Strategies to Overcome Stability Challenges of Single-Atom Nanozymes

**DOI:** 10.1007/s40820-025-01939-2

**Published:** 2026-01-05

**Authors:** Rong Guo, Qiuzheng Du, Yaping He, Haoan Wu, Yu Zhang, Ziwei Jing

**Affiliations:** 1 Department of Pharmacy, The First Affiliated Hospital of Zhengzhou University, Zhengzhou, 450000 People’s Republic of China; 2 State Key Laboratory of Digital Medical Engineering, Jiangsu Key Laboratory for Biomaterials and Devices, School of Biological Science and Medical Engineering and Basic Medicine Research and Innovation Center of Ministry of Education, Zhongda Hospital, Southeast University, Nanjing, 211102 People’s Republic of China

**Keywords:** Single-atom nanozymes, Clinical translation, Stability issues, Innovative strategies, Biocompatibility

## Abstract

Single-atom nanozymes (SAzymes) exhibit exceptional catalytic efficiency due to their maximized atom utilization and precisely modulated metal-carrier interactions, which have attracted significant attention in the biomedical field. However, stability issues may impede the clinical translation of SAzymes. This review provides a comprehensive overview of the applications of SAzymes in various biomedical fields, including disease diagnosis (e.g., biosensors and diagnostic imaging), antitumor therapy (e.g., photothermal therapy, photodynamic therapy, sonodynamic therapy, and immunotherapy), antimicrobial therapy, and anti-oxidative stress therapy. More importantly, the existing challenges of SAzymes are discussed, such as metal atom clustering and active site loss, ligand bond breakage at high temperature, insufficient environment tolerance, biosecurity risks, and limited catalytic long-term stability. Finally, several innovative strategies to address these stability concerns are proposed—synthesis process optimization (space-limited strategy, coordination site design, bimetallic synergistic strategy, defect engineering strategy, atom stripping-capture), surface modification, and dynamic responsive design—that collectively pave the way for robust, clinically viable SAzymes.

## Highlights

- This review uniquely provides an in-depth focus on the stability issues of single-atom nanozymes (SAzymes), covering multiple aspects such as metal atom clustering and active site loss, ligand bond breakage at high temperature, insufficient environment tolerance, biosecurity risks, and limited catalytic long-term stability.
- This review integrates and systematically discusses a wide range of potential strategies to overcome stability issues, including synthesis process optimization (space-limited strategy, coordination site design, bimetallic synergistic strategy, defect engineering strategy, atom stripping-capture), surface modification, and dynamic responsive design.
- To transform SAzymes from “star materials” of the laboratory into precise clinical tools for medicine, the authors propose the four-dimensional roadmap: structure-predictable, activity-tunable, biocompatible, and scalable.

## Graphical abstract

**Figure:**
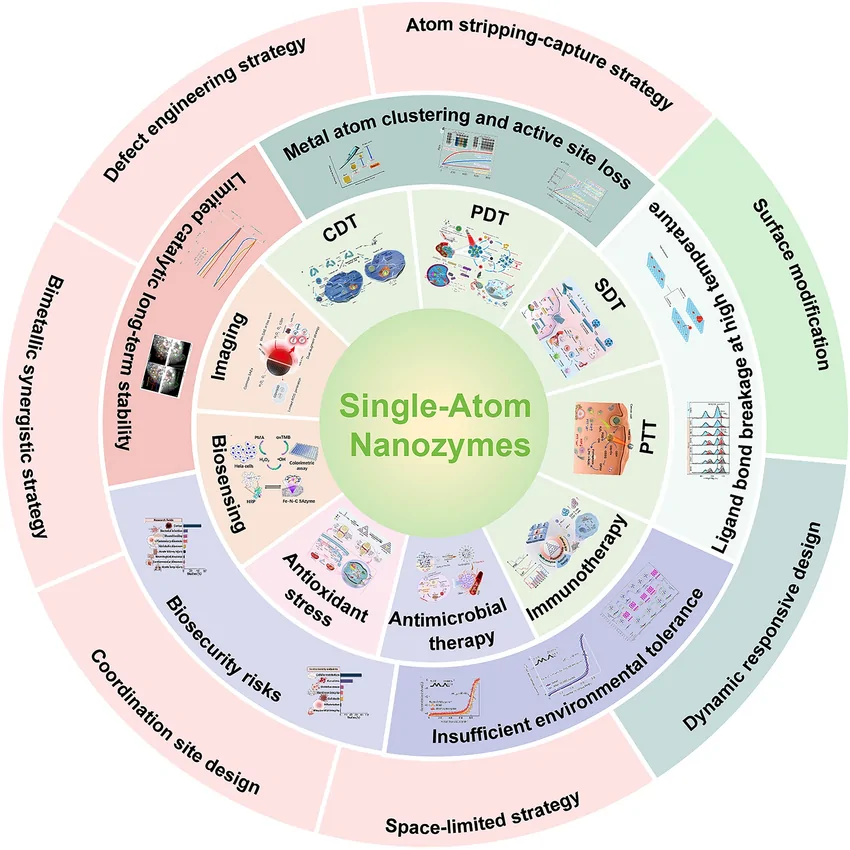

## Introduction

The field of nanozymes experienced a significant milestone in 2007 when Yan et al. first demonstrated that Fe_3_O_4_ nanoparticles exhibit natural horseradish peroxidase activity, a discovery that generated substantial scientific interest [1]. Building on this foundation, Wei and Wang provided a defining characterization in 2013, introducing nanozymes as “a class of mimetic enzymes that combine the unique properties of nanomaterials with distinctive catalytic functions” [2]. In 2022, our research team established the first standardized development process for prussian blue nanozymes [3], and further elucidated the catalytic mechanism of Fe_3_O_4_ nanoparticles. Specifically, we revealed that Fe^2+^ ions within the Fe_3_O_4_ structure can regenerate surface Fe^2+^ through electron transfer mediated by the Fe^2+^–O–Fe^3+^ chain, enabling sustained POD-like catalytic activity. This cyclic process allows for continuous regeneration of surface Fe^2+^ ions, maintaining prolonged catalytic function [4]. To expand the practical applications of these findings, our research group collaborated with Xu to spearhead the development of China’s inaugural international standard within ISO/TC229/WG3 published in 2023, the standard titled “Nanotechnology-Methods for measuring peroxidase-like activity of metal and metal oxide nanoparticles” (ISO/TS 5094: 2023) provides critical methodology for evaluating nanozyme activity [5]. Collectively, these advancements have transformed the understanding of inorganic nanomaterials, moving beyond the traditional view of their biological inertness to reveal their intrinsic biological effects and novel properties. This paradigm shift has significantly broadened the scope of research in the field of mimetic enzymes, extending from organic complexes to inorganic nanomaterials, and has opened new avenues for innovative biomedical applications.

While traditional nanozymes offer significant benefit, such as high stability, low cost, large-scale production, designability, and multifunctional integration, they are still hindered by several critical limitations. These include a low density of active sites, insufficient electron transfer efficiency, and relatively low catalytic efficiency [6, 7]. The multilevel structural features of nanozymes result in a complex catalytic mechanism [8], making it challenging for conventional nanozymes to accurately replicate the intricate coordination structure and catalytic microenvironment of natural enzyme active centers. This limitation leads to compromised catalytic selectivity and activity [9]. In contrast, single-atom nanozymes (SAzymes) provide an innovative solution to overcome these limitations. By anchoring metal atoms in an isolated form on the carrier surface, SAzymes address the issues of dispersed active sites and low atom utilization commonly seen in traditional nanozymes. For instance, Fe-N_4_Cl SAzymes have been optimized through axial chlorination engineering, achieving a POD activity 4.9 times higher than conventional SAzymes [10]. Furthermore, RhN_4_ and VN_4_ SAzymes demonstrate a catalytic affinity exceeding that of natural enzymes by 5–20 times due to their unique dual reaction pathways. These SAzymes also exhibit stability over months and can be reused dozens of times [11]. Beyond these advantages, SAzymes show remarkable potential in the biomedical field. By maximizing atom utilization and precisely modulating metal-carrier interactions, SAzymes combine high catalytic efficiency—for example, exhibiting laccase-like activity up to 4.72 times that of natural enzymes [7]—with multi-scenario adaptability [12, 13]. These advancements not only bridge the performance gap between natural enzymes and artificial catalysts but also provide a theoretical framework for biomimetic catalytic design at the atomic scale [14, 15].

The synthesis of SAzymes typically employs methods such as high-temperature pyrolysis, wet chemical approach, and atomic layer deposition (ALD) [16]. In a typical pyrolysis process, the SAzymes are formed by decomposition of metal precursors at high temperatures to disperse isolated atoms onto vacancies and defects within the support framework, the entire process is generally carried out under an inert atmosphere [17]. Wet chemical approaches rely on solvents reducing metal precursors to single atom metals and then keeping them uniformly dispersed on the support. Defective supports supply abundant anchoring sites to trap isolated metal atoms [6]. ALD is a thin film deposition method that conducted under inert atmospheres, vacuum, or high temperature. Through continuous and self-limiting reactions between the gaseous precursors and substrate, it enables a straight forward approach for the homogeneous deposition of atoms on high surface area substrates [18]. In addition to the above three synthesis methods, other approaches such as one-pot high-temperature calcination method, salt-template method, thermal emission and adsorption method have been also reported to construct SAzymes. While these methods could successfully engineer atomically dispersed, highly active sites (e.g., M-N_4_), yet they are ultimately constrained by intrinsically weak metal-carriers bonding, dynamic passivation of anchoring defects, and process-induced instabilities.

**Fig. 1:**
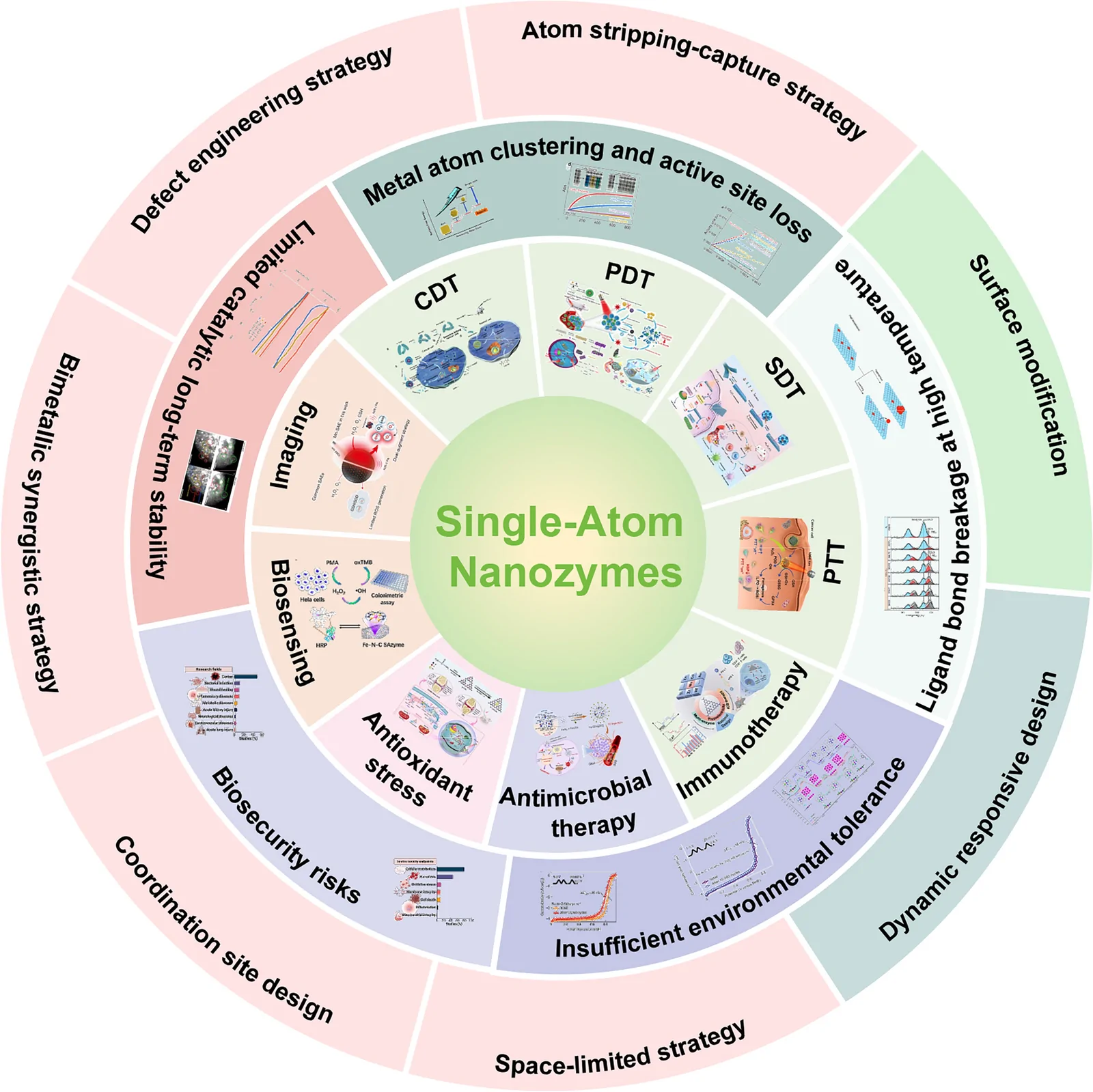
Schematic illustration of the innovative strategies to address the stability challenges of single-atom nanozymes

This review provides a comprehensive overview of the applications of SAzymes in the biomedical field, including their utility in disease diagnosis (e.g., biosensors and diagnostic imaging), antitumor therapy (e.g., photothermal therapy, photodynamic therapy, sonodynamic therapy, immunotherapy), antimicrobial therapy, and oxidative stress mitigation. More importantly, it further summarize the stability issues of SAzymes, such as metal atom clustering and active site loss, ligand bond breakage at high temperature, insufficient environment tolerance, biosecurity risks, and limited catalytic long-term stability. Finally, the review proposes targeted strategies to address these stability concerns. These include enhancing metal-carrier interactions through defect engineering, ligand site design, and spatial confinement strategies. Additionally, surface modification and dynamic responsive design approaches are suggested to minimize the nonspecific distribution of SAzymes and enhance their biocompatibility (Fig. 1).

## Biomedical Applications of SAzymes

### Disease Diagnosis

SAzymes have shown remarkable performance in disease diagnosis, exhibiting high sensitivity, selectivity, and stability. These characteristics enable them to accommodate a broad spectrum of biological environments and diagnostic requirements. Their capability in detecting small molecules like nitric oxide (NO), hydrogen sulfide (H_2_S), dopamine (DA), and uric acid (UA) is noteworthy. Moreover, SAzymes enable precise imaging of lesion sites, establishing them as multimodal platforms for biomedical applications [19]. Collectively, these advances underscore SAzymes' substantial promise in biomedicine, particularly for early disease detection and real-time monitoring.

#### Biosensing Detection

**Fig. 2:**
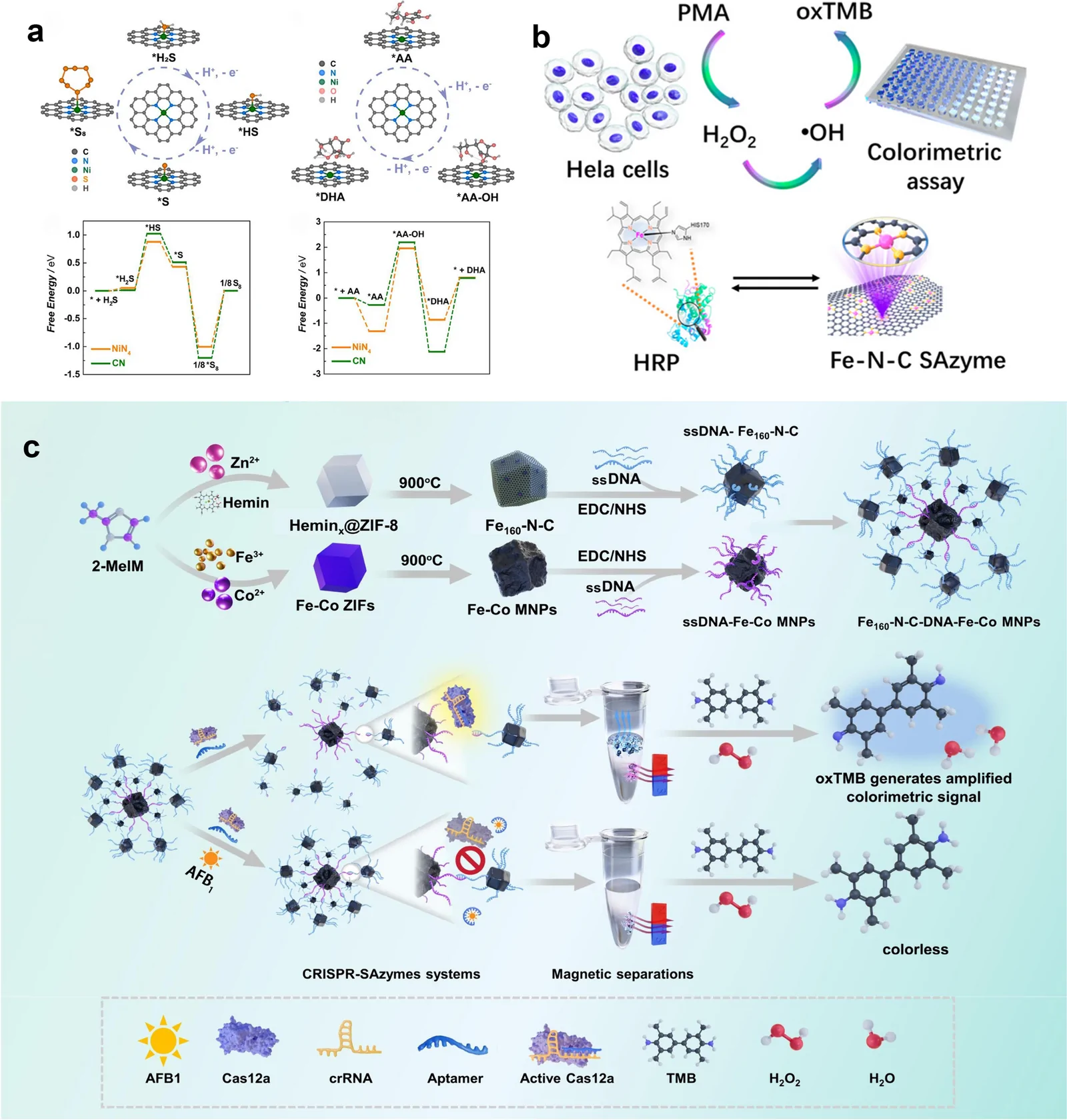
**a** Mechanism of NiN_4_-SAC-catalyzed selective H_2_S oxidation [21]. Copyright 2022, American Chemical Society. **b** Mechanism of H_2_O_2_ Detection Released from HeLa Cells by Fe–N-C SAzyme [22]. Copyright 2019, American Chemical Society. **c** Synthesis and modification of Fe–N-C SAzymes and Fe–Co MNPs, the reaction principle for CRISPR-SAzymes sensors [25]. Copyright 2024, ScienceDirect

Biosensing detection applications of SAzymes leverage their high catalytic activity and specificity to construct sensitive biosensors for biomarker detection. For instance, Chen et al. developed iron-based SAzymes systems, including Fe–N–C SAN and Fe–N/C, which enable efficient detection of butyrylcholinesterase (BChE) and alkaline phosphatase (ALP). Their smartphone-integrated paper-based bioassay allows convenient BChE activity monitoring, while Fe–N/C achieves highly sensitive ALP activity screening [20]. Meanwhile, Ni-SACs combined with electrochemical redox (GRP) technology enable the detection of H₂S release behavior, particularly in mouse brain applications, achieving high sensitivity with strong interference resistance (Fig. 2a) [21]. Additionally, CNT/FeNC and Fe–N–C, based on iron atoms anchored on N-doped carbon nanotubes, facilitate highly sensitive glucose and ascorbic acid detection. These systems achieve target molecule detection through H_2_O_2_ activation or (glucose oxidase) GOx-mediated cascade reactions, with corresponding visual biosensors developed (Fig. 2b) [22]. Ru-SACs have been applied for simultaneous detection of dopamine (DA) and uric acid (UA), attaining high sensitivity and excellent selectivity by adjusting the electrode response potential difference to 180 mV [23]. Xie et al. established an ALP activity detection strategy using the Fe/NC-SA system. In this approach, TMB is oxidized to a blue color by Fe/NC-SAs in the presence of H_2_O_2_. The blue oxTMB color fades in the presence of ascorbic acid (AA), which can be hydrolyzed from ascorbic acid 2-phosphate (AAP) by ALP. This colorimetric assay demonstrates high sensitivity, good selectivity, and strong anti-interference properties for ALP activity analysis and has been successfully validated using human serum samples [24]. Furthermore, Liu et al. constructed a colorimetric sensor based on the CRISPR/Cas12a system, utilizing Fe–N-C SAzymes and Fe–Co magnetic nanoparticles for aflatoxin B_1_ detection. The system releases Fe–N–C nanozymes through the CRISPR/Cas12a cleavage reaction, enabling TMB substrate oxidation and detection via colorimetric changes (Fig. 2c) [25]. Generally, the above-mentioned Fe/Ni/Ru–N–C SAzymes used in biosensing suffer from several stability shortcomings, such as pyrolysis-induced framework collapse (> 700 ℃), metal leaching induced by physiological environments, high ion/thiol competitive coordination, ROS self-erosion, and storage inactivation. These shortcomings may lead to a 40–60% decrease in activity within 20 days, false positives caused by leached ions, and the need for daily calibration for continuous monitoring.

#### Diagnostic Imaging

**Fig. 3:**
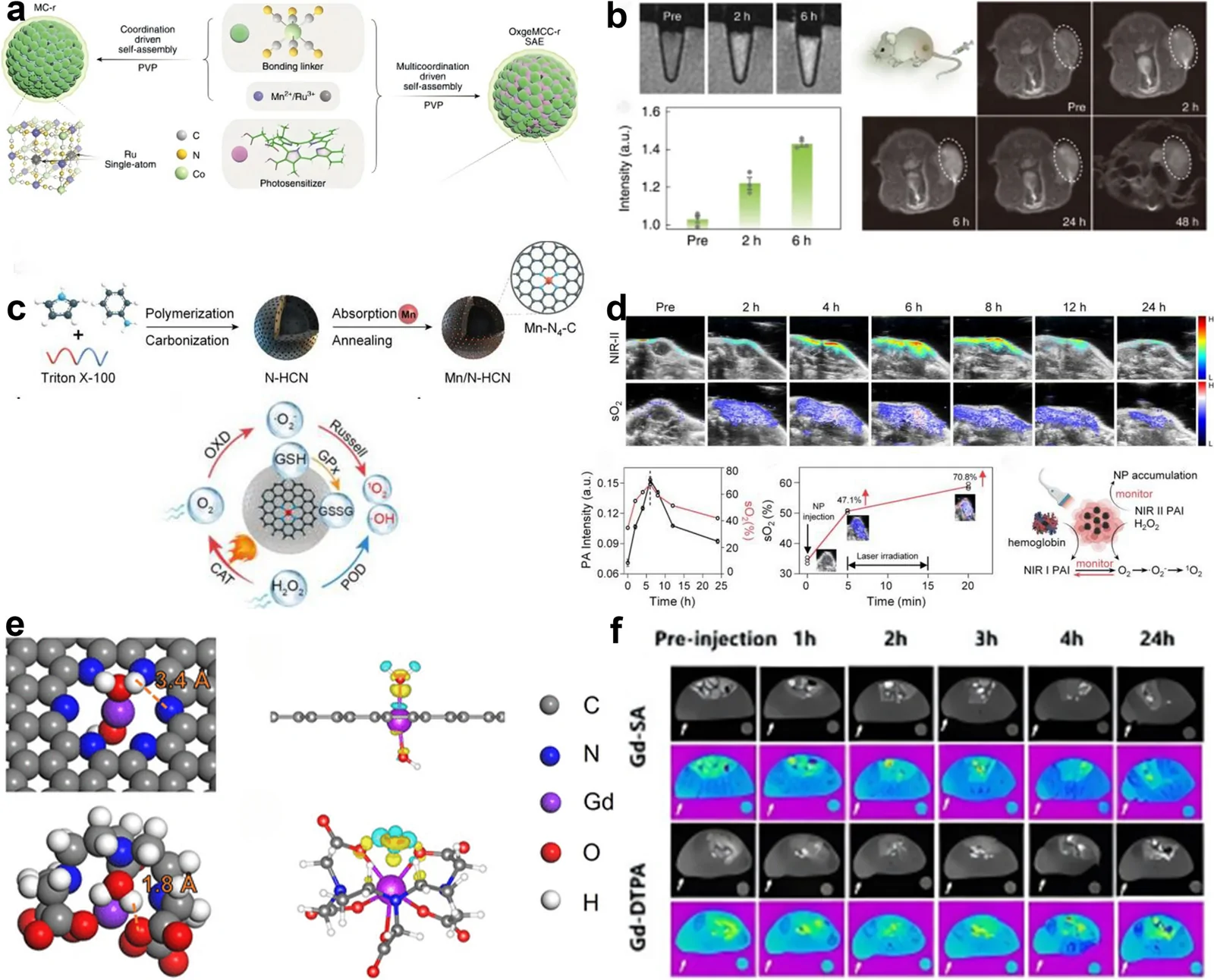
**a** Schematic illustration of OxgeMCC-r. OxgeMCC-r consists of catalytically active single-atom Ru site anchored in MCC with outer PVP protection layer [27]. Copyright 2020, Springer Nature. **b** Corresponding relative MR imaging intensity, and in vivo T_1_-weighted magnetic resonance images of 4T1 tumor-bearing mouse at various time points post-injection. Tumor regions are marked with white dashed lines [27]. Copyright 2020, Springer Nature. **c** Schematic illustration of coupled multi-nanocatalytic activities of Mn/N-HCN in tumor tissue [29]. Copyright 2023, Springer Nature. **d** Intra-tumor PA imaging of Mn/N-HCN and SO_2_ level after intravenous injection of Mn/N-HCN, and change of sO_2_ level after intratumoral injection of Mn/N-HCN and 1064 nm laser irradiation [29]. Copyright 2023, Springer Nature. **e** DFT studies on the transfer of H_2_O molecules on Gd-SA and Gd-DTPA [30]. Copyright 2023, American Chemical Society. **f** *T*_1_-weighted normal tumor (50–150 mm^3^) MR images of tumor-bearing BALB/c mice before and after intravenous injection of Gd-SA and Gd-DTPA at different time points under a 7 T magnetic field [30]. Copyright 2023, American Chemical Society

The imaging properties of SAzymes can be optimized for targeted applications in magnetic resonance imaging (MRI), fluorescence, and photothermal imaging through the strategic selection of metal species (e.g., Mn, Fe, Co, Cu) and modulation of their coordination environments (e.g., M-N_4_ structures) [26]. For example, Wang et al. developed the OxgeMCC-r SAE system, which leverages the coordination of Mn with six nitrogen atoms to form a high-spin structure, endowing it with MRI capabilities. In tumor-specific MRI tests, this system achieved maximum signal intensity within six hours of intravenous administration and maintained robust signaling even after 48 h, highlighting its potential for extended MRI-guided in vivo therapeutic applications (Fig. 3a, b) [27]. In another study, Chang et al. loaded Cu-SAzymes with luseogliflozin and employed a 1064 nm laser to induce photothermal effects, generating localized high temperatures for controlled drug release. The strong near-infrared absorption of the black carbon carrier facilitated photothermal imaging (PTI), enabling real-time monitoring of temperature changes in the tumor region and assessment of therapeutic efficacy [28]. Wang et al. also constructed Mn-SAE nanozymes with NIR-II window responsiveness properties suitable for near-infrared second window (NIR-II) photoacoustic imaging and photothermal therapy. These nanozymes exhibited significantly enhanced catalase-like (CAT) activity. Photoacoustic imaging was used to monitor the biodistribution, accumulation kinetics in tumor tissues, and in situ catalytic activity of the nanozymes, providing guidance for optimizing combination therapies (Fig. 3c, d) [29]. Additionally, Liu et al. introduced a single-atom Gd-based nanocontrast agent (Gd-SA) designed to enhance tumor MRI. Gd-SA features a large surface area and rapid relaxation of water molecules, achieving superior T_1_-weighted MRI enhancement at 7 T. Compared to Gd-DTPA, in vivo MRI results demonstrated that Gd-SA offers higher spatial resolution and a broader time window for tumor imaging, along with favorable biocompatibility (Fig. 3e, f) [30]. However, SAzymes such as Ru-, Cu-, Mn- and Gd-SAzymes may encounter issues such as metal-ion leaching, oxidative aggregation or reduction-induced detachment, carrier degradation in acidic/reductive tumor milieus, and batch-to-batch inconsistency after storage.

### Disease Treatment

#### Tumor Treatment

The tumor microenvironment is defined by several key features, including hypoxia, low pH, elevated levels of reactive oxygen species (ROS), an abundant extracellular matrix, and the presence of immunosuppressive cells [31]. These characteristics create a unique environment that can be exploited for therapeutic purposes. SAzymes, leveraging their diverse enzymatic activities, have shown significant potential in cancer therapy. They can harness the acidic conditions and endogenous H_2_O_2_ within the tumor microenvironment to mediate cancer cell death through various therapeutic modalities, such as chemodynamic therapy (CDT), photodynamic therapy (PDT), sonodynamic therapy (SDT), and photothermal therapy (PTT) [32, 33]. Beyond this, SAzymes also demonstrate an ability to disrupt tumor metabolic pathways, thereby cutting off the energy supply to tumors and triggering cell death. Additionally, they can remodel the immune microenvironment, enhancing the overall efficacy of immunotherapeutic approaches (Table 1) [34].

**Table 1:** A summary of enhanced antitumor therapy models based on TME-activated enzymatic activities of SAzymes

Sazymes	Coordination condition	Metal, loading (wt%)	Enzymatic activities	Therapy models	Refs
OxgeMCC-r SAE	Ru-C_6_	Ru, 2.23	POD,CAT	PDT	[27]
SAF NPs@DOX @CM	/	Fe, /	POD	CDT	[35]
Mn-N/C	Mn-N_4_	Mn, /	POD	CDT, ICD	[36]
C_3_N_4_-Mn SAC	Mn-C_3_N_4_	Mn, 2.34	GSHOx, enhanced absorption in the red-light region	PDT	[37]
HNCDs	Fe-N_4_	Fe, /	type I photodynamic activity, red fluorescence emission	PDT, Pyroptosis	[38]
BP@Au@FA-PEG	/	Au, /	POD	PDT	[39]
OxgeMCC-r SAE	Ru-C_6_	Ru, 2.23	POD, CAT	PDT	[27]
Fe-C₃N_4_ NS	Fe-C_3_N_4_	Fe, 0.16	POD	CDT, SDT	[40]
PdSA/Ti_3−*x*_C_2_T_y_	Pd-C_3_	Pd, 2.5	POD, CAT	SDT, ICD	[41]
Zn/Pt SATs	/	Zn, 0.3 Pt, 1.0	POD, GSHOx	SDT, Ferroptosis	[42]
SAFe-NMCNs	Fe-N_4_	Fe, 1.85	POD, CAT	PTT	[43]
Pd SAzyme	Pd-N_4_	Pd, 0.18	POD, GSHOx	PTT, Ferroptosis	[44]
CeSAs	/	Ce, 1.18	POD, CAT, GOD, GSH-Px	PTT	[45]
Mo SAs	Mo-C_3_N_4_	Mo, 0.71	POD, CAT	ICD, ICB	[46]
Cu-NS@UK@POx	Cu-N_3_S_1_	Cu, /	POD, NOx, LCO, GSHOx	Pyroptosis,	[47]
SeSAE	Se-NC	Se, /	POD, GSHOx, NOx	Other therapies	[48]
M/GLB@AuSAN	Au-N_3_	Au, /	CAT, POD, OXD, NADHox	Ferroptosis, Apoptosis	[49]
Fe-CDs	Fe-N_4_	Fe, 3.4	CAT, SOD, OXD, POD, GSH-Px, TPx	Apoptosis	[50]

##### Chemodynamic Therapy (CDT)

Some SAzymes can induce the production of a significant quantity of hydroxyl radicals (·OH), known for their potent oxidative properties, within tumor cells. This is achieved via the Fenton reaction or a Fenton-like reaction [51]. These hydroxyl radicals target critical biological macromolecules in tumor cells, including DNA, proteins, and lipids, thereby triggering tumor cell apoptosis and accomplishing tumor treatment goals. Beyond this, SAzymes are capable of generating oxygen through catalytic reactions, which helps to alleviate the hypoxic conditions often present in the tumor microenvironment and consequently enhances the efficacy of chemodynamic therapy. For example, Liu et al. developed a porous iron-based SAzyme (SAF NPs@DOX@CM), which exhibits POD-like activity and effectively initiates a tumor-specific Fenton reaction in situ. This reaction selectively produces abundant ·OH radicals under the acidic conditions of the TME. Moreover, after being modified with CM, SAF NPs@DOX@CM can achieve homotypic binding with target tumor tissues through homologous binding, preventing premature clearance. This system enhances cancer therapy by combining the functions of drug carriers with enzyme therapy at single-atom catalytic sites [35]. In another study, Qiao et al. constructed a manganese-based SAzyme (Mn-N/C), which catalyzes the conversion of cytosolic H_2_O_2_ into ·OH radicals via a Fenton-like reaction. This process generates sufficient ROS to induce immunogenic cell death (ICD) in tumor cells, thereby significantly boosting anti-tumor immunity mediated by CD^8+^ T cells [36] (Table 2).

**Table 2:** A summary of SAzymes Based on Multiple Synthesis Strategies to Enhance Stability

SAzymes	Coordination condition	Metal loading (wt%)	Synthesis process	Refs
PMCS	Zn-N_4_	Zn, 3.12	Space-limited strategy	[72]
CuN_3_-SAzyme	Cu-N_3_	Cu, 2.98	Space-limited strategy	[89]
Co-SAEs/HNCS	Co-N_4_	Co, 0.30	Space-limited strategy	[90]
FeN_5_ SAzyme	Fe-N_5_	Fe, /	Space-limited strategy	[91]
Cu-CN	Cu-N_4_	Cu, 23.36	Space-limited strategy	[92]
Fe-B/N–C SAzymes	Fe-N_4_	Fe, /	Coordination site design strategy	[94]
H-MoN_5_@PtN_4_/C	Pt-N_4_	Pt, /	Coordination site design strategy	[95]
Fe-NC	Fe_1_-NS_1.3_C	Fe, /	Coordination site design strategy	[100]
PEG@P@Ce-N/S-C	Ce-N_4_S_2_	Ce, 2.47	Coordination site design strategy	[102]
FeSNC	Fe-N_3_S_1_	Fe, 1.3	Coordination site design strategy	[101]
FeN_3_P-SAzyme	Fe-N_3_P	Fe, /	Coordination site design strategy	[83]
20Pt/SGCN-500	Pt₁-N₃PS	Pt, 20.6	Coordination site design strategy	[105]
MIrPHE	Ir-N_4_	Ir, 5.45	Coordination site design strategy	[96]
Zn/Mo DSAC-SMA	Zn-C_1_N_3_-O-Mo-N_4_	Zn, 1.5 Mo, 7.3	Bimetallic synergistic strategy	[107]
FeCu-DA	Fe-N_2_C_2_-Cu-N_2_C_1_	Fe, 0.8 Cu, 0.4	Bimetallic synergistic strategy	[108]
Pt_NPs_-Fe/NC	Fe-N_4_	Pt, 1.28 Fe, 0.49	Bimetallic synergistic strategy	[109]
FePc-15/N-C	Fe-N_4_	Fe, 0.45	Defect Engineering Strategy	[111]
2Cu/CeO_2_-800@GOx	Cu-O_3_	Cu, /	Defect Engineering Strategy	[112]
Pt_TS_-SAzyme	Pt-N_3_PS	Pt, 0.07	Atom Stripping-Capture Strategy	[77]

##### Photodynamic Therapy (PDT)

PDT leverages the photosensitizer-integrated and catalytic activity of nanozymes. When SAzymes are irradiated with light at specific wavelengths, they generate substantial ROS, which induce tumor cell apoptosis or necrosis [52]. For example, Yin et al. reported that in the tumor microenvironment, Mn atoms can be incorporated into the cavities of C₃N_4_ nanosheets to form Mn-C_3_N_4_ SAC. This structure enhances red light absorption through a ligand-to-metal charge transfer process. Under 660 nm irradiation, it induces hydrolysis to produce O_2_-independent ·OH, enabling tumor-specific PDT [37]. Li et al. developed red-light-excited photosensitive carbon nanodots (HNCDs), which inherit the monatomic Fe-N_4_ center of heme chloride. By creating a *sp*^2^ hybridized carbon environment, they converted energy transfer to electron transfer, efficiently inducing tumor cell death and activating anti-tumor immune responses even under anoxic conditions. This approach effectively inhibits the growth and metastasis of triple-negative breast cancer [38]. Zhu et al. constructed a PEGylated BP@Au@FA nanoplatform (BP@Au@FA-PEG), which significantly enhanced the therapeutic effect on hepatocellular carcinoma (HCC) via PDT. Under near-infrared (NIR) irradiation, it efficiently generates ROS, including single-linear oxygen (^1^O_2_) and hydroxyl radicals (·OH), thereby effectively killing tumor cells [39]. Wang et al. designed a single-atom ruthenium nanoenzyme (OxgeMCC-r) by immobilizing single-atom ruthenium in a Mn_3_[Co(CN)_6_]_2_ MOF material containing the photosensitizer dihydroporphyrin (Ce6). Under endogenous H_2_O_2_ conditions, OxgeMCC-r exhibits CAT activity to produce O_2_, alleviating the hypoxia-attenuated therapeutic effect of PDT [27].

##### Sonodynamic Therapy (SDT)

SDT leverages ultrasound to activate the sound-sensitive properties of nanozymes, generating ROS that can damage tumor cell membranes, DNA, and other structures, thereby inducing apoptosis or necrosis [53]. For example, Feng et al. designed a Fe-doped SAzymes graphitic phase carbon nitride (C_3_N_4_) semiconductor nanosheets (Fe-C_3_N_4_ NS) as chemically active sonosensitizers. Not only did single-atom Fe doping drastically improve the separation efficiency of e⁻-h⁺ pairs in SDT, both in vitro and in vivo experiments demonstrated that Fe-C_3_N_4_ NS possessed an excellent antitumor effect through the enhancement of acoustic chemical kinetic effects [40]. Geng et al. utilized the abundant Ti vacancies on the surface of Ti_3−*x*_C_2_T_y_ nanosheets to reduce and stabilize the Pd single atoms, with the loading of single-atom Pd as high as 2.5 wt%, and obtained Pd single-atom dispersed Ti_3−*x*_C_2_T_y_ nanosheets (PdSA/Ti_3−*x*_C_2_T_y_), which were tested for in vitro acoustic kinetic activity and found that the Pd atoms could act as electron traps, effectively preventing the excited The compounding of electron–hole pairs significantly enhances the acoustic kinetic activity, while at the same time, the holes generated under ultrasound irradiation can rapidly consume the overexpressed glutathione in the tumor microenvironment, further enhancing the efficiency of reactive oxygen species generation [41]. Wen et al. developed an oxygen-defect-rich Zn/Pt dual-site single-atom-loaded TiO_2_ sonosensitizers (Zn/Pt SATs) for Zn/Pt-driven bifunctional stacking-enhanced SDT, where the presence of Zn improves the electron transport capacity of the system, while the electrons in the conduction band of the 5*d* orbitals of Pt are activated, which further improves the electronic excitation efficiency and the activation of O_2_ and H_2_O efficiency, thus effectively promoting the separation of electron–hole pairs and realizing efficient SDT performance [42].

##### Photothermal Therapy (PTT)

Some SAzymes can efficiently convert light energy into heat energy under near-infrared light irradiation, which increases the temperature of tumor tissues and induces hyperthermia-induced ablation to tumor cells, enabling photothermal therapy [54]. Su et al. designed SAFe-NMCNs with POD and CAT, which can convert H_2_O_2_ and generate a large amount of ·OH and O_2_ in the tumor microenvironment; moreover, SAFe-NMCNs exhibit significant light absorption in the second-order near-infrared (NIR-II) biological window, and when subjected to 1064 nm laser light irradiation, they are able to effectively convert light energy into thermal energy, significantly enhancing the local temperature. This photothermal effect further enhanced the catalytic activity of the nanoenzymes and accelerated the generation of ·OH radicals, which resulted in a more intense killing effect on cancer cells. The significant anti-tumor effect of SAFe-NMCNs nanoenzymes under the synergistic effect of photothermal and nanocatalysis was verified by in vitro and in vivo experiments [43]. Chang et al. prepared a nitrogen-liganded carbon-supported Pd SAzyme exhibiting dual POD and GSHOx activities, along with photothermal conversion capabilities. This SAzyme induces ferroptosis and shows excellent NIR-II (1000–1400 nm) photothermal properties. DFT mechanistic studies further revealed that under tumor microenvironment (TME) conditions, the Pd SAzyme maintains high catalytic activity at safe therapeutic temperatures (42 ± 0.5 °C), significantly enhancing ROS generation in cancer cells [55]. Guo et al. prepared cerium SAzymes (CeSAs), and the reversible conversion between Ce^3+^ and Ce^4+^ endowed the CeSAs with multi-enzyme cascade activities, such as POD activity, CAT activity, GOD activity and GSH-Px activity. Meanwhile, the prepared CeSAs also possessed photothermal properties, which could realize photothermal therapy (PTT) of tumor cells under 808 nm near infrared (NIR) irradiation, and the catalytic-photothermal synergy of the multi-enzyme activities and photothermal properties of CeSAs significantly enhanced the tumor therapeutic effect of CeSAs [45].

##### Immunotherapy

Tumor immunotherapy refers to exogenous modulation in the body’s immune system, reactivate the cancer-immunity cycle, restoring and improving the body’s anti-tumor immune response, so as to achieve the therapeutic effect of controlling or even specifically eliminating tumors [56]. At the same time, nanoenzymes combined with other immunotherapies can further improve the therapeutic effect and reduce the adverse effects on the basis of improving the tumor microenvironment and enhancing the immune response. Currently, the main methods of nanoenzymes combined with immunotherapy include: immune checkpoint inhibitor combination therapy, CAR-T cell therapy combination therapy, bispecific antibody combination therapy, tumor vaccine combination therapy, relay cell therapy combination therapy and so on [57]. For example, Lin et al. developed a molybdenum SAzymes (Mo SAs) capable of exerting potent therapeutic effects on immune checkpoint blockade (ICB)-resistant tumors [46], Mo SAs can remodel the tumor immune microenvironment by inducing tumor immunogenic cell death, alleviating tumor hypoxia, and modulating tumor chemokine expression, which further enhances the anti-tumor efficacy with anti-PD-L1 therapy and highlights its potential for treating ICB-resistant tumors. Niu et al. developed a novel SAzymes inducer, i.e., Cu-NS SAzymes (Cu-NS@UK@POx) coloaded with UK5099 and pyruvate oxidase (SpxB), which can trigger scavenging via cascade biocatalysis to enhance the immunogenicity of tumor cells, and target pyruvate metabolism to remodel the immunosuppressive tumor microenvironment. By inducing the generation of reactive oxygen storm, depletion of NADH/glutathione/L-cysteine, pyruvate oxidation, and lactate/ATP depletion, it triggers cell death and metabolic modulation, and significantly activates anti-tumor immunotherapy [47].

##### Other Therapies

Cheng et al. developed a non-metallic selenium SAzymes (SeSAE) with nicotinamide adenine dinucleotide phosphate (NADPH)-like oxidase activity, which has a favorable biosafety profile, efficiently converts NADPH to NADP^+^ and induces O_2_^·−^ production. In addition, SeSAE exhibited potent ROS generation and NADPH depletion in mouse tumor cells, leading to severe oxidative stress and disruption of cellular energy metabolism, thus overcoming the resistance of tumor cells to catalytic therapy [48]. Wang et al. developed a highly ordered biomimetic composite nanoenzyme M/GLB@AuSAN, in which loaded GOx and (lactate oxidase) LOx aerobically catalyzed the generation of H_2_O_2_ from glucose and lactic acid, and H_2_O_2_ was rapidly converted to ·OH, O_2_^·−^, and O_2_ via AuSAN, and the generated O_2_ served as a positive feedback for further GOx- and LOx-mediated aerobic catalysis. These substrates are catalytically converted, significantly amplifying cascade reactions and enhancing ROS accumulation to effectively inhibit melanoma progression [49]. Ultrasmall carbon dot-loaded iron SAzymes (Fe-CDs) prepared by Muhammad et al. possessed six enzymatic activities, CAT, SOD, OXD, POD, GSH-Px, and thiol peroxidase (TPx), while BBB-permeable and glioma-targeting peptides were introduced on Fe-CDs for selective glioblastoma (GBM) targeting in vivo. The results showed that the cascade enzyme activities of Fe-CDs stimulated autophagy, which effectively inhibited tumor growth in a drug-resistant GBM mouse model [50].

#### Antimicrobial Therapy

**Fig. 4:**
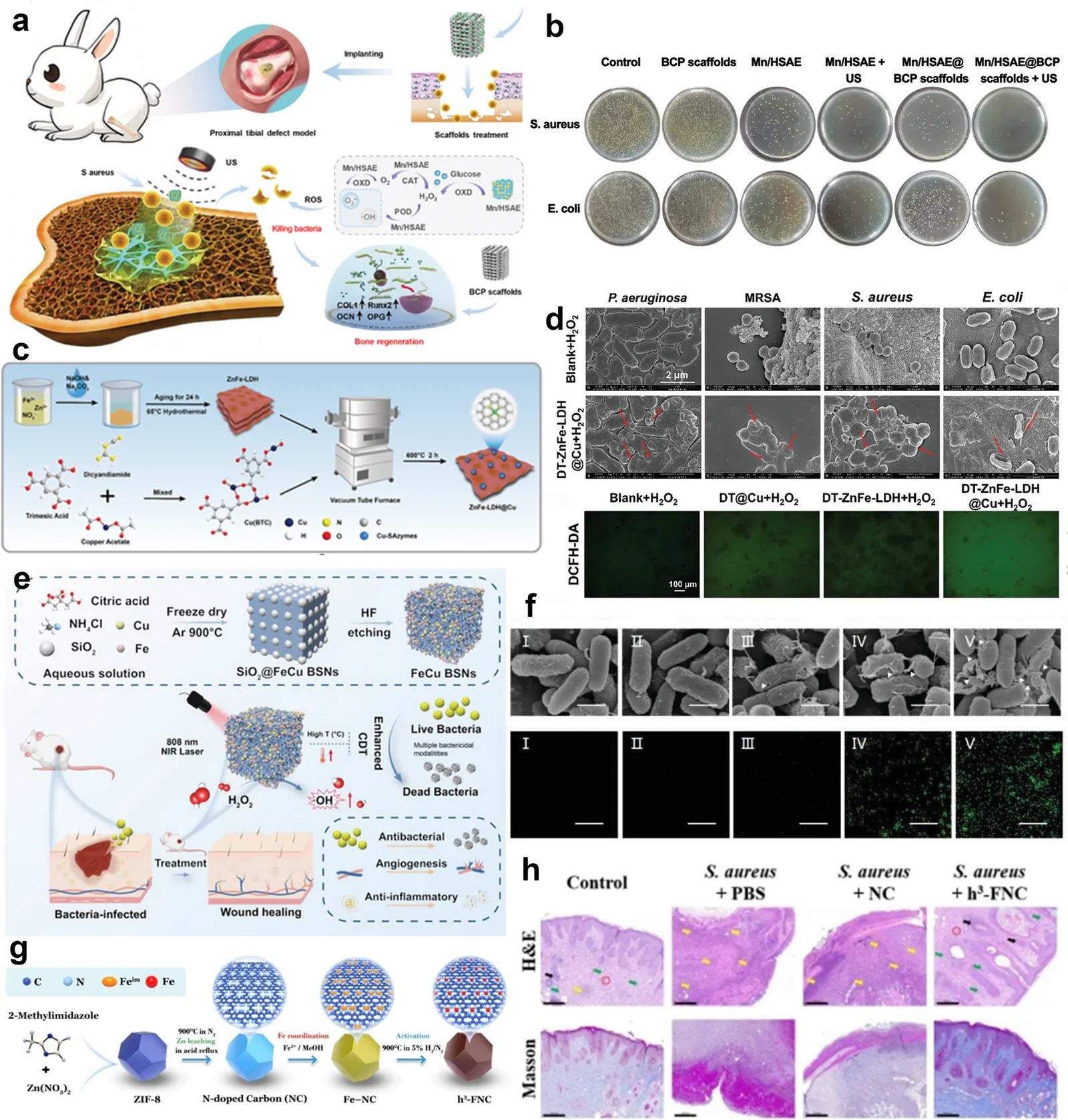
**a** Mn/HSAE@BCP scaffolds treatment for rabbit model of infective tibial bone defect [58]. Copyright 2024, Wiley. **b** Representative photographs of proliferation of *S. aureus* and *Escherichia coli* (*E. coli*) after coculture with BCP scaffolds, Mn/HSAE (55 µg/mL), Mn/HSAE + US, Mn/HSAE@BCP scaffolds, Mn/HSAE@BCP scaffolds + US [58]. Copyright 2024, Wiley. **c** Schematic illustration of the manufacturing process of ZnFe-LDH@Cu [59]. Copyright 2025, Wiley. **d** SEM images visualizing morphology of bacteria incubated with different treatment groups, and ROS levels in *P. aeruginosa* were monitored by DCFH-DA after different treatments [59]. Copyright 2025, Wiley. **e** Schematic illustration of FeCu BSNs for efficient PTT-CDT antibacterial and wound healing [60]. Copyright 2024, Wiley. **f** SEM images of *P. aeruginosa* under various treatments, and fluorescence images of *P. aeruginosa* stained with DCFH-DA [60]. Copyright 2024, Wiley. **g** Synthetic method for h^3^-FNCs with high Fe loading [61]. Copyright 2025, Springer Nature. **h** H&E and Masson staining of the bacteria-infected tissues after different treatments. Yellow arrows reflected inflammatory cells, including macrophages, lymphocytes, and neutrophils. Green and black arrows represent fibroblasts and hair follicles, respectively. Red circles represent neovascularization [61]. Copyright 2025, Springer Nature

Some SAzymes have good antimicrobial properties, which can destroy the cell membrane, DNA and other structures of bacteria by generating reactive oxygen species to achieve the effect of killing bacteria, which is important for the treatment of bacterial infectious diseases. Our group prepared a 3D-printed Mn/HSAE@BCP scaffold with functionalized modification of manganese SAzymes, which can catalyze the generation of ·OH and O_2_^·−^ from H_2_O_2_ through a cascade reaction, and is able to generate abundant ROS to kill bacteria. In addition, the enhanced antibacterial effect of Mn/HSAE@BCP scaffold facilitates the expression of osteogenic inducing factors in vitro, further promoting bone regeneration in vivo. (Fig. 4a, b) [58]. DT-ZnFe-LDH@Cu is a copper SAzymes loaded by ZnFe layered double hydroxide. Under acidic conditions, the nanoenzyme exhibits POD and OXD activities and is capable of catalyzing the generation of a variety of reactive oxygen species (e.g., ·OH, O_2_^·−^, and ^1^O_2_) for antimicrobial therapy. Under neutral and alkaline conditions, its CAT activity converts excess ROS to oxygen, relieves hypoxia and modulates inflammatory responses (Fig. 4c, d) [59]. Zhai et al. developed a bimetallic SAzyme (FeCu BSNs) composed of iron and copper, which exhibited excellent photothermal conversion efficiency (56.26%) and POD activity (752.25 U mg^−1^). In the combined application of photothermal therapy (PTT) and chemodynamic therapy (CDT), FeCu BSNs significantly enhanced antimicrobial efficacy and wound healing by generating high levels of ROS and localized thermal effects. In addition, their low dose (10 μg mL^−1^) can achieve efficient antimicrobial and tissue repair capabilities (Fig. 4e, f) [60]. The iron SAC (h^3^-FNC) developed by Chen et al. exhibits exceptional oxidase-like catalytic performance, featuring a high metal loading of 6.27 wt% and an optimized adjacent Fe distance of approximately 4 Å. This catalyst effectively facilitates ROS generation and GSH depletion, leading to membrane disruption, DNA damage, and protein leakage in infected areas. Consequently, it demonstrates potent eradication of both Staphylococcus aureus and Pseudomonas aeruginosa in vitro and in vivo. Notably, the catalyst maintains robust antibacterial efficacy without significant activity degradation after six months of storage (Fig. 4g, h) [61].

#### Antioxidant Stress Therapy

**Fig. 5:**
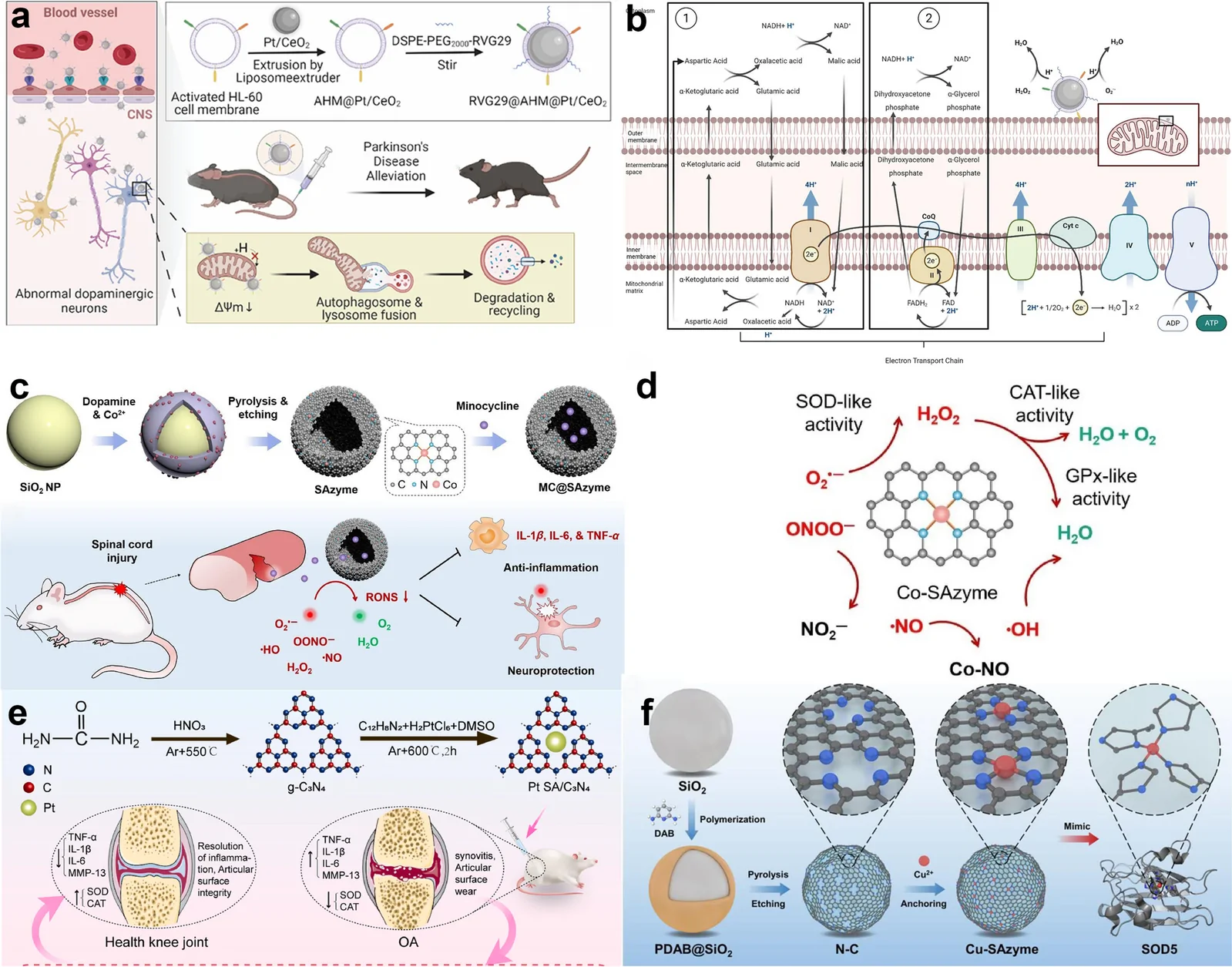
**a** Synthesis, modification, and preparation of core–shell structured single-atom catalysts and the mechanism of inducing abnormal mitophagy, thus improving Parkinson’s motor and nonmotor symptoms [62]. Copyright 2023, American Chemical Society. **b** Exploration of the mechanism of mitophagy induced by single-atom catalysts Pt/CeO_2_ [62]. Copyright 2023, American Chemical Society. **c** Single-atom Co nanozyme promoted the recovery of spinal cord injury [63]. Copyright 2023, Springer Nature. **d** Illustration of the possible mechanisms of RONS scavenging for Co-SAzyme [63]. Copyright 2023, Springer Nature. **e** Manufacture of Pt SA/C_3_N_4_ nanozymes and the fundamentals of biomimetic SOD and CAT for the scavenging of ROS [64]. Copyright 2024, ScienceDirect. **f** Post-adsorption strategy to mimic the microenvironment of SOD5. DAB, 2,6-diaminopyridine. PDAB, polymer of 2,6-diaminopyridine [65]. Copyright 2022, Wiley

Oxidative stress refers to the imbalance of oxidation and antioxidant balance in the body, resulting in excessive production of free radicals such as reactive oxygen species (ROS) or reactive nitrogen species (RNS), which exceeds the body’s own antioxidant capacity, thus triggering damage to cells, tissues, and organs, and is closely related to the onset and development of a variety of diseases. For example, the main pathogenesis of PD, a neurodegenerative disease, involves damage and dysfunction of dopaminergic neurons, in which oxidative stress due to ROS is a key factor. Li et al. designed a novel Sazymes (Pt/CeO_2_) that indirectly triggers self-service of dysfunctional mitochondria by interfering with the α-glycerophosphate shuttle pathway and malate-aspartate shuttle pathway clearance to treat Parkinson’s disease (PD) (Fig. 5a, b) [62]. Oxidative stress and inflammation are the major pathophysiological processes in traumatic spinal cord injury (SCI), and a SAzyme (Co-SAzyme) synthesized by Jiang et al. with a hollow structure can reduce RONS and inflammation in secondary spinal cord injuries. Co-SAzyme showed the ability to eliminate overexpression of H_2_O_2_, O_2_^·−^, ·OH, nitric oxide (NO), and peroxynitrite (ONOO^−^) in the early stage of SCI.Meanwhile, minocycline encapsulated in a porous hollow structure is released continuously for synergistic neuroprotective effects (Fig. 5c, d) [63]. Osteoarthritis is caused by the overproduction of ROS and RNS and abnormal ATPmetabolism related to oxidative phosphorylation pathway in mitochondria. Xiang et al. prepared Pt SAzymes (Pt SA/C_3_N_4_) loaded with g-C_3_N_4_, which have SOD and CAT activities can scavenge ROS/RNS and regulate mitochondrial ATP production, helping to reverse the oxidative stress-induced articular cartilage damage and slow down the progression of osteoarthritis (Fig. 5e) [64]. Sepsis is a severe life-threatening systemic inflammatory response syndrome caused by microbial infections. In the pathogenesis, electron leakage from the ubiquinone site of the mitochondrial respiratory chain generates a large amount of O_2_^·−^, which destroys the cells and tissues, and may ultimately lead to infectious shock and multi-organ dysfunction syndrome. Yang et al. synthesized Cu-SAzyme featuring divalent Cu–N_4_ centers that exhibit high SOD-like activity, markedly reducing oxidative DNA damage and the secretion of pro-inflammatory cytokines (Fig. 5f) [65]. The core causative factor of dry eye disease (DED) is inflammation, in which ROS play a key role in the vicious cycle of DED by regulating upstream inflammation. Zhu et al. developed a novel eye drop based on dual-atom nanozymes (DANs), which was successfully prepared by embedding Fe and Mn bimetallic single-atoms was successfully prepared by embedding Fe and Mn bimetallic single atoms in N-doped carbon materials and modified with hydrophilic polymers. In vitro and in vivo results showed that DAN possessed excellent bioactivities in scavenging excessive ROS, inhibiting the activation of NLRP3 inflammatory vesicles, decreasing the expression of pro-inflammatory cytokines, and inhibiting cell apoptosis [66]. Kurian et al. designed NZ-engineered hydrogels (NZ@hydrogels) that precisely modulate ROS levels to target a variety of injuries and pathologic dermatoses [67].

## Challenges in the Stability Issues

### Metal Atom Clustering and Active Site Loss

**Fig. 6:**
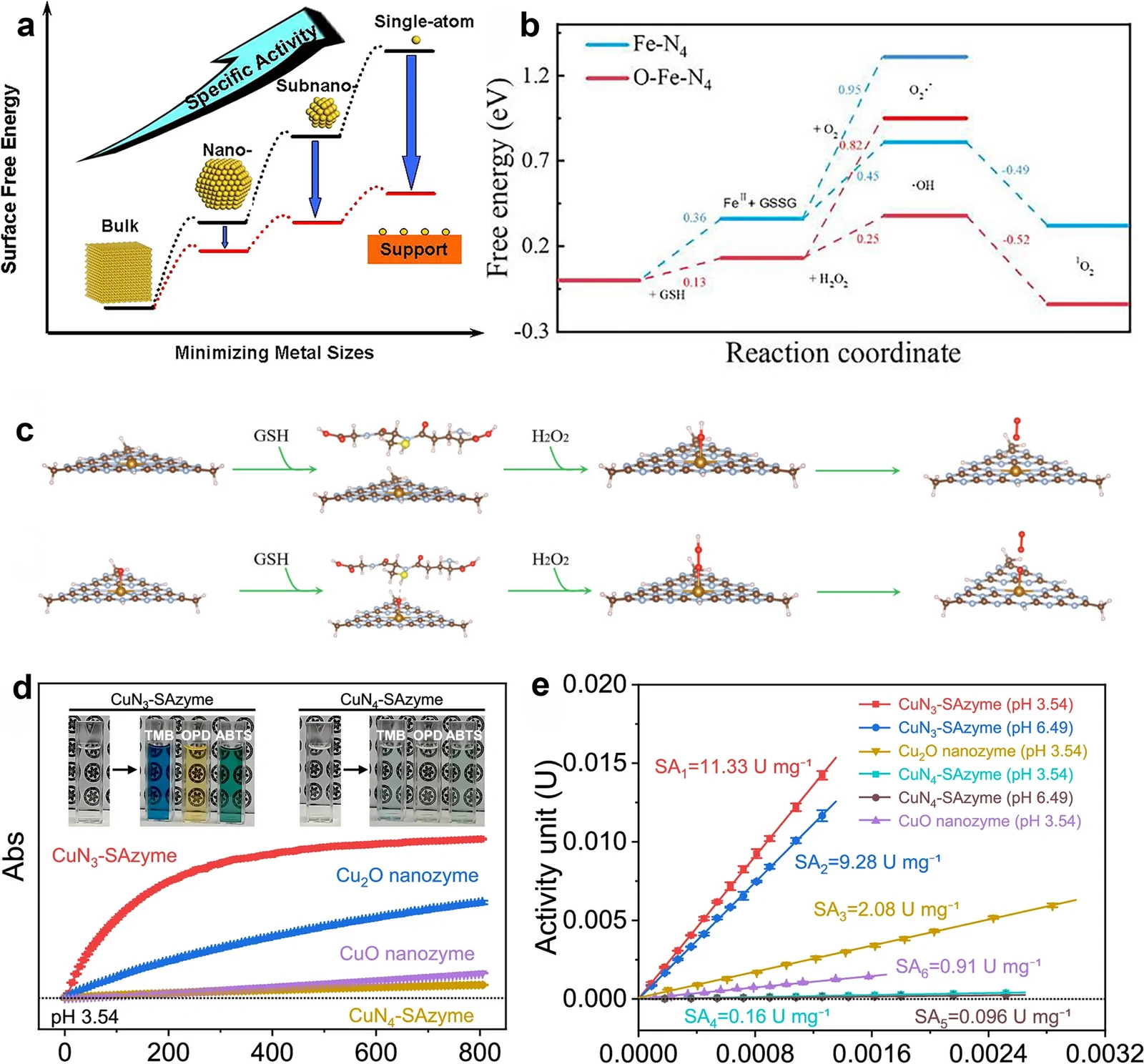
**a** Schematic illustrate the changes of surface free energy and specific activity per metal atom with metal particle size and the support effects on stabilizing single atoms [70]. Copyright 2024, American Chemical Society. **b** Corresponding free energy diagram [71]. Copyright 2024, Wiley. **c** The proposed catalytic mechanism for Russell reaction on FeN_4_ moiety and O-FeN_4_ moiet [71]. Copyright 2024, Wiley. **d** Reaction-time curves of TMB colorimetric reaction catalyzed by CuN_*x*_-SAzymes and CuO_*x*_ nanozymes. Inset: Photographs of peroxidase substrate (TMB, OPD, and ABTS) solutions catalyzed by CuN_*x*_-SAzymes [72]. Copyright 2024, Springer Nature. **e** Comparison of the specific activities (U/mg) of CuN_*x*_-SAzymes and CuO_*x*_ nanozymes [72]. Copyright 2024, Springer Nature

The metal active sites of SAzymes are susceptible to migration and agglomeration during catalytic reactions or long-term storage due to their high surface free energy, which results in decreased activity [68]. Metal active sites of SAzymes (e.g., Fe, Co, etc.) have high surface free energies due to atomic-level dispersion. This thermodynamic instability promotes the migration and agglomeration of isolated metal atoms into nanoparticles or clusters during catalytic reactions or storage, leading to a decrease in active site density and attenuation of catalytic performance (Fig. 6a) [69, 70]. For example, Liu et al. found that the active sites of conventional SAzymes (e.g., Fe-N_4_) usually have a symmetric electronic structure, resulting in a high activation energy of the catalytic reaction pathway, which limits the selectivity and generation efficiency of ^1^O_2_. This symmetry makes it difficult to optimize the adsorption and desorption processes of the reaction intermediates, thus reducing the catalytic activity (Fig. 6b, c) [71].

Meanwhile, Luo et al. mentioned that when the metal loading of SAzymes is too high, the interactions between the metal atoms are enhanced beyond the threshold of the anchoring capacity of the carriers, leading to migration of the atoms and formation of nanoparticles [69]. In addition, the high-temperature carbonization and solvent treatment steps involved in the synthesis process may disrupt the metal-carrier coordination structure, further exacerbating the risk of agglomeration. When SAzymes are agglomerated, some metal atoms will gather to form metal clusters or particles, and the interactions between these agglomerated metal atoms will change their electronic structures and coordination environments, whereas the activity of SAzymes mainly originates from the interactions between their individual metal atoms and the carriers, as well as the coordination environments of the metal atoms, and thus agglomeration will result in the change or disappearance of the original active sites, and the active sites on the surface of the agglomerated SAzymes, the active sites on their surfaces are partially blocked or covered, and it is difficult for the substrate molecules to effectively adsorb onto the active sites, thus hindering the catalytic reaction. Not only that, agglomeration also affects its stability and service life, and further structural changes and deactivation are more likely to occur during the catalytic reaction, leading to the shortening of its stability and service life in practical applications. For example, Wu et al. mentioned that the Cu-N_4_ coordination structure in CuN_4_-SAzymes is susceptible to H_2_O_2_ adsorption during the catalytic process, which leads to the loss of active sites and consequently to a decrease in catalytic efficiency (Fig. 6d, e) [72].

### Ligand Bond Breakage at High Temperature

**Fig. 7:**
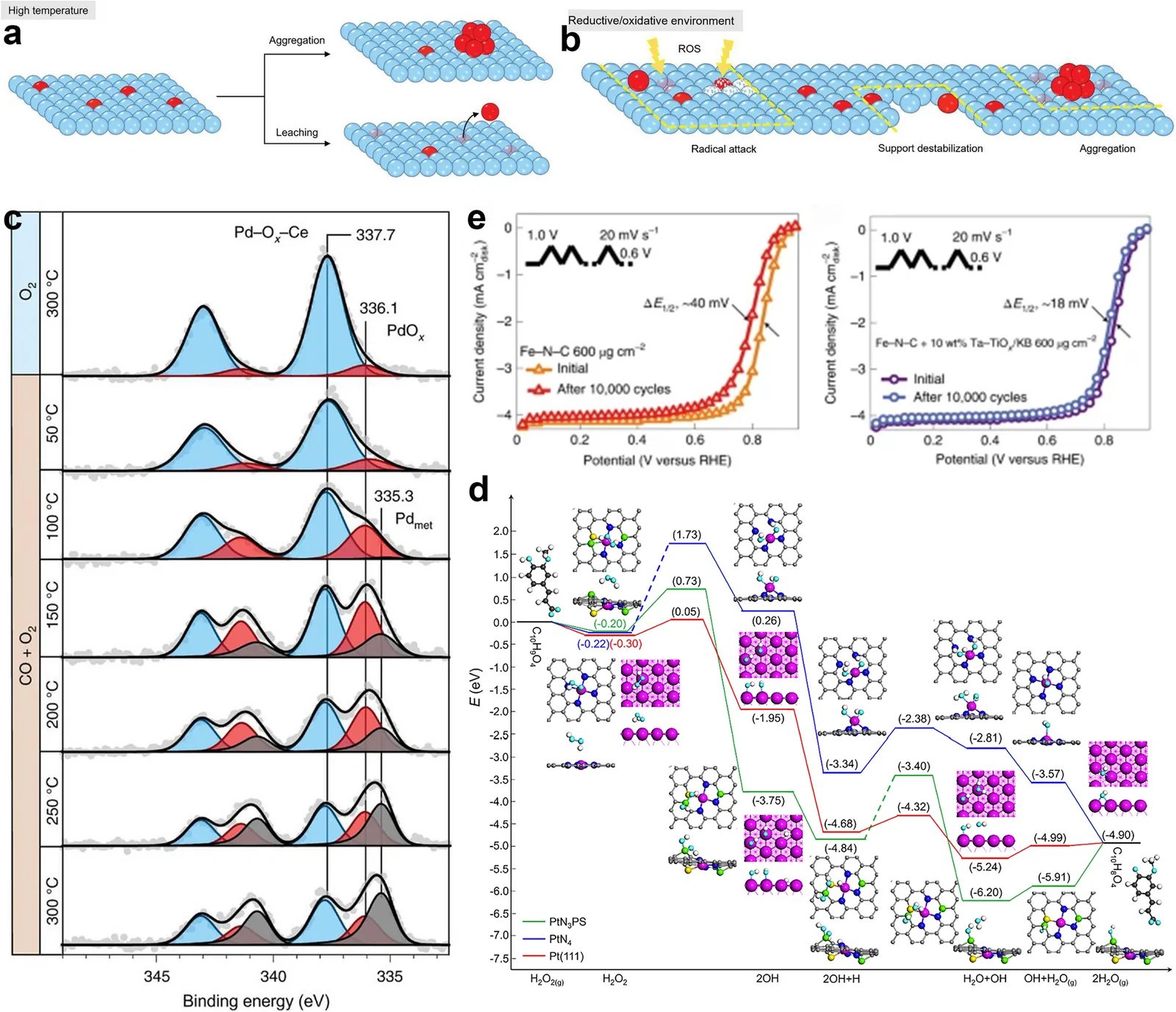
**a** Schematic of high temperature-induced SAzymes deactivation, including atom aggregation and leaching [73]. Copyright 2025, Wiley. **b** Schematic of SAzymes deactivation under reductive/oxidative conditions, including radical attack, substrate destabilization, and aggregation [73]. Copyright 2025, Wiley. **c** Extensive reduction process of 5PdRods in CO oxidation under high temperatures is characterized by in situ NAP-XPS. Reproduced with permission [75]. Copyright 2021, Springer Nature. **d** DFT studies on the peroxidase-like activity of PtN_3_PS and PtN_4_ SAzymes, as well as Pt NPs. In the energy profiles, the most favorable paths of H_2_O_2_ dissociation into surface ·OH species in the acidic condition, as well as the oxidation reaction of TMB in thermodynamically are shown. The Pt, C, N, P, S, O and H atoms are given in pink, gray, blue, green, yellow, cyan and white, respectively; while in order to make a distinction, the C atoms in TMB are shown in dark gray [77]. Copyright 2021, American Chemical Society. **e** The RRDE durability test of the Fe–N–C catalyst without the scavengers, showing the ORR performance at the initial cycle and after 10,000 potential cycles, and the durability test of the Fe–N–C catalyst with the scavengers (10 wt%, Ta to Ti ratio = 6:4) [78]. Copyright 2022, Springer Nature

SAzymes can break ligand bonds in high-temperature environments, thus weakening metal-carrier interactions and leading to detachment or agglomeration of metal atoms. The main reason for the limited stability of SAzymes at elevated temperatures is the increased kinetic energy of the metal atoms at high temperatures, which allows them to overcome diffusion barriers more easily. This accelerates their migration and aggregation, which leads to a significant reduction of active sites and thus affects their catalytic efficiency (Fig. 7a, b) [73]. Yang et al. reported that Pd SACs remained stable at 100 °C but exhibited a significant loss of activity at 600 °C [74]. Muravev et al. observed that the temperature-dependent behavior of Pd in 5PdFSP and 5PdRods SACs during CO oxidation reactions (Fig. 7c) [75]. Zhong et al. found that metal atoms tend to aggregate to form nanoparticles in single-atom catalysts at high temperatures or in reducing e atmospheres [76].

### Insufficient Environment Tolerance

The metal active sites of SAzymes are anchored to carriers (e.g., carbon-based materials, MOFs) via ligand bonds, Metal-N/C/O ligand bonds are weakened when the metal’s d-orbitals become electron-deficient in strong acids, bases, or highly oxidizing media, lowering bond dissociation energies and promoting atomic detachment. Simultaneously, corrosion of carbon or MOF scaffolds exposes coordination vacancies, removing the physical anchors that maintain atomic dispersion. This triggers dynamic restructuring: single-atom sites can reversibly transform into lower-coordination motifs or agglomerate into clusters, a process accelerated by radical attack on coordinating N atoms and by excessive H_2_O_2_ that oxidizes the metal center. Chen et al. showed that under high H_2_O_2_ concentration or low pH conditions, the coordination structure of the Pt monoatom may undergo aberrations, leading to a deviation of the catalytic pathway from the expected one, or even to the generation of low-activity products, such as H_2_O (Fig. 7d) [77]. Fe–N–C SAzymes are susceptible to free radical attack in acidic media, leading to detachment of Fe atoms from the coordination site and reducing their durability, but the durability of Fe–N–C SAzymes was significantly enhanced in the presence of free radical scavengers (Fig. 7e) [78]. For SAzymes, the coordination environment of the active site (e.g., the type and number of coordinating atoms) affects their chemical stability and catalytic activity. For example, Wei et al. pointed out that Fe-N_4_ nanoenzymes without the introduction of axial Cl coordination are prone to structural distortions in high H_2_O_2_ concentration environments, limiting their long-term stability [10].

### Biosecurity Risks

**Fig. 8:**
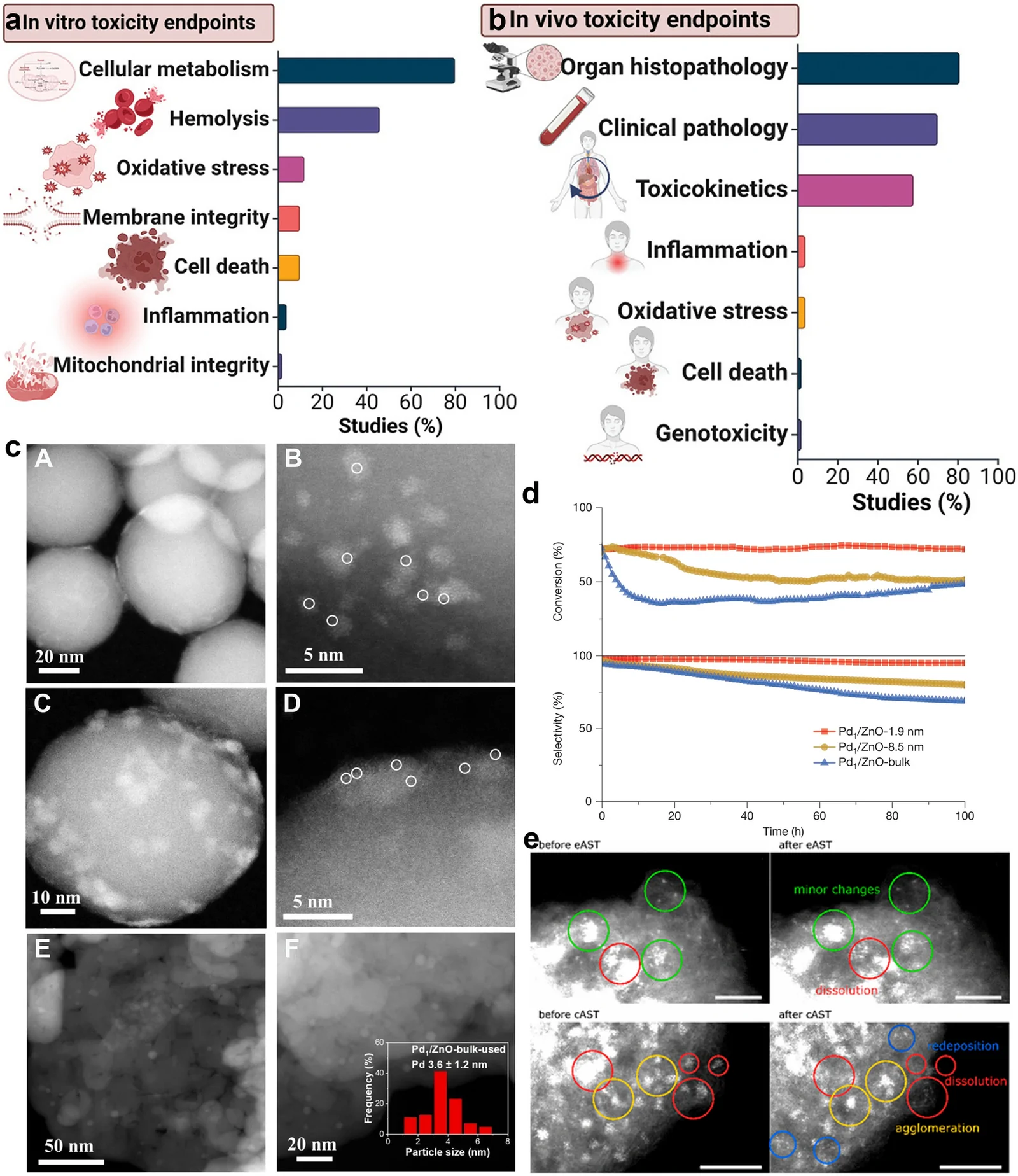
**a** In vitro toxicological endpoints addressed in scientific studies evaluating the therapeutic potential of nanozymes [79]. Copyright 2024, Wiley. **b** In vivo toxicological endpoints addressed in scientific studies evaluating the therapeutic potential of nanozymes [79]. Copyright 2024, Wiley. **c** Morphology of Pd1/ZnO-ynm catalysts after the long-term stability test in acetylene hydrogenation, representative HAADF-STEM images of Pd1/ZnO-1.9 nm (**A**, **B**), Pd1/ZnO-8.5 nm (**C**, **D**), and of Pd1/ZnO-bulk (**E**, **F**) at low and high magnifications. The white circles in (**B**, **D**) highlight Pd single atom and the inset in f represent the Pd size distributions of Pd1/ZnO-bulk-used [84]. Copyright 2025, Springer Nature. **d** Acetylene conversion and ethylene selectivity as a function of time during long-term stability tests of Pd_1_/ZnO-1.9 nm, Pd_1_/ZnO-8.5 nm and Pd_1_/ZnO-bulk [84]. Copyright 2025, Springer Nature. **e** HAADF IL-TEM images before and after 1000 cycles of an eAST and cAST in 0.1 M HClO_4_. Green denotes the spots of interest where only minor changes are observed, red denotes dissolution, yellow denotes the spots of interest where agglomeration is observed, and blue denotes the appearance of new single atoms [82]. Copyright 2020, American Chemical Society

Non-degradable carrier materials (e.g., traditional carbon-based materials) may remain in the body for a long time, triggering inflammation or immune responses. The long-term toxicity of SAzymes in the body is still unclear, and further studies on their metabolic processes and long-term effects in the body are needed. Meanwhile, once nanomedicines enter the bloodstream, they may interact with different blood components (e.g., plasma proteins and blood cells), thereby interfering with their physiological functions and posing a threat to normal body physiology (Fig. 8a, b) [79]. For example, Sun et al. found that nanoparticles (NPs) pose a physiological threat by impeding the coagulation system, leading to NP-induced coagulopathy and affecting hemostatic homeostasis [80]. In addition, in the environment of strong acid, strong alkali or highly oxidizing medium, the ligand bonds of SAzymes may be broken, the carrier structure is corroded, leading to the detachment of metal atoms, and the chronic leakage of metal ions (e.g., Co, Ir) may lead to the cumulative toxicity in organs, and the SAzymes of noble metals (e.g., Pt, Ir), though with high catalytic efficiency, may trigger cytotoxicity at high doses. Moreover, SAzymes lack protective mechanisms in organisms compared to natural enzymes [15], such as protein folding and modification, which makes SAzymes more susceptible to damage in complex chemical environments. Furthermore, the substrate specificity of SAzymes is usually low, and they are susceptible to interference by other substances, leading to decreased catalytic selectivity [81]. In living organisms, a variety of biomolecules and ions are present, which may bind non-specifically to SAzymes and affect their catalytic properties.

### Limited Catalytic Long-term Stability

The high free energy of the metal surface of SAzymes easily causes the aggregation of active sites, which is prone to agglomeration during prolonged catalysis and storage, and SAzymes lack the protection mechanisms of natural enzymes, which makes them more susceptible to inactivation by external factors during the storage and catalysis process. For example, Cherevko et al. proposed a mechanism for the inactivation of Pt SAC, which was investigated over six consecutive cycles in 0.1 M HClO using a combination of in situ ICP-MS, IL-TEM, and XPS, and the anodic peaks of the Pt SAC segregated more and more intensely as the cycles were repeated, suggesting progressive clustering of the Pt SAC, which intensified inactivation. Meanwhile, HAADF-STEM imaging revealed that the Pd1/ZnO-bulk used exhibited severe aggregation (Fig. 8c, d) [82]. The unoptimized Fe-N_4_ SAzyme showed a significant decrease in POD activity after 8 weeks of storage at room temperature, which was mainly attributed to the gradual migration and agglomeration of Fe single atoms into Fe nanoparticles due to the high surface free energy, resulting in a decrease in the density of active sites [83]. Shi et al. found that in stability tests, conventional bulk-phase ZnO-loaded Pd SACs showed reduced catalytic selectivity and severe metal aggregation (Fig. 8e) [84].

## Potential Strategies to Overcome Stability Issues

### Synthesis Process Optimization

The challenge in the preparation of SAzymes lies in the effective prevention of spontaneous agglomeration of isolated metal atoms intoNPs with relatively low surface free energies [85]. Therefore, constructing strong covalent coordination interactions between the central metal single-atom and the surrounding coordination atoms to reduce the specific surface energy of the metal single-atom sites is essential for the synthesis of stable single-atom catalysts [86–88].

#### Space-limited Strategy

The spatial domain-limiting strategy utilizes the pores of some materials as molecular cages to spatially segregate metal atoms, achieve atomic-level dispersion of metal species, and prevent them from migrating and agglomerating. The protocol comprises two steps. First, the pore structure of the porous material is used to separate and encapsulate the mononuclear metal precursor with suitable size to realize the spatial isolation and atomic level dispersion of the metal species. Subsequently, in the post-processing of removing the ligands of the metal precursor to form the single atoms, the metal single atoms formed by using the ligands anchored to the ligand sites on the porous material itself or on the material derived from it are selected to be able to select the carriers with strong confinement ability to effectively prevent the metal atoms from agglomerating. The choice of a carrier with strong domain-limiting ability can effectively prevent the metal atoms from agglomerating, and at the same time, different carrier materials can also give the SAzymes diverse catalytic properties. For example, the researchers designed and synthesized a highly active SAzyme containing Zn–porphyrin structure by utilizing metal–organic framework (MOF) material ZIF-8 as a precursor and a mesoporous silica-protected strategy modeled after the natural peroxidase (HRP), and the bacterial inhibition rate remained more than 95% after 6 months of storage [89]. Wu et al. regulated the coordination environment of copper atoms by anchoring Cu single atoms to two-dimensional carbon nanostructures, and CuN_3_-SAzyme not only exhibited higher POD activity than CuN_4_-SAzyme, but also maintained high enzyme activity without any significant changes in the geometry and coordination environment after a total dose of 500 Gy of radiation [72]. Dai et al. formed Co-SAEs/HNCS by anchoring Co single atoms to HNCS substrate, which provided a stable support and optimized coordination environment to enhance the stability of Co single atoms, thus improving the stability and catalytic performance of Co-SAEs/HNCS [90]. Xu et al. prepared a five-coordinated monoatomic iron SAzyme Fe-N_5_ via a ZIF-8 template, in which ZIF-8, as a zinc-based MOF, provided a stable coordination environment for the iron monoatoms and optimized their electronic structure, and the POD activity of the monoatomic iron SAzymes Fe-N_5_ was enhanced by 3.45 × 10^5^-fold compared with that of the Fe_3_O_4_ SAzymes, and it exhibited enhanced anti-tumor effects both in vivo and ex vivo effects in vivo and in vitro [91].

In addition, nitrogen-containing carbonyl (C_3_N_4_) carriers were formed during high-temperature carbonization through formamide as a carbon source and reducing agent, in which the high nitrogen content and abundant vacant coordination sites of formamide can effectively chelate Cu^2+^ and reduce it to CuI during carbonization to form atomically dispersed active sites. This strategy realizes the homogeneous anchoring of CuI monoatoms in a one-step method and avoids the problem of metal agglomeration in the traditional multi-step synthesis. Liu et al. prepared copper SAzymes with an ultra-high atomic density of 23.36 wt% by the above method, and the experimental results showed that Cu-CN has the potential for excellent cascade catalytic activity and good biocompatibility in both in vivo and ex vivo experiments [92].

#### Coordination Site Design Strategy

Metal-carrier interactions can change the electronic structure and surface properties of catalysts, thus affecting their catalytic performance and stability [93], the ligand design strategy enhances metal-carrier interactions by rationally designing ligand sites on carriers that can anchor metal precursors or metal atoms [70], in order to stabilize the metal single atoms and prevent them from migrating and agglomerating, and achieve the synthesis of SAzymes.

On the one hand, SAzyme stability is enhanced by modifying the electronic structure and binding energy through axial coordination modifications, introducing axial coordination atoms (e.g., O, Cl, S) to modulate the electronic structure of the metal active site. For example, Liu et al. effectively modulated the local coordination environment of planar Fe-N_4_ motifs (Fe-B/N–C SAzymes) by spatially axial boron (B) ligands. Through electronic modulation, Fe-B/N–C SAzymes exhibited significantly enhanced OXD, POD, and CAT activities. Theoretical calculations showed that the spatially axial B ligand effectively modulated the charge distribution around the planar Fe-N_4_ active center, which favored heterolytic cleavage and desorption of H_2_O_2_, thus accelerating the decomposition of H_2_O_2_. Meanwhile, the spatially axial B ligand enhanced the metal-carrier interactions and improved the stability of the Fe-B/N–C SAzymes [94]. A hollow axial Mo-Pt SAzyme (H-MoN_5_@PtN_4_/C) was constructed using a two-layer template capture strategy, in which the axial ligand induces Mo 4d orbital splitting leading to a spin-electron rearrangement that modulates the enzyme activity, generates CAT activity and enhances OXD activity, and, on the basis of overcoming the anaerobic negative effects of the tumors, further enriches the enriched cytotoxic superoxide radicals (O_2_^·−^). Notably, H-MoN_5_@PtN_4_/C exhibited a destructive d–π conjugation between the active center and the substrate, attenuating orbital and electronic confinement and significantly enhancing the enzyme-like activities (POD and OXD) of the Mo monoatom and the POD performance of the Pt monoatom. In addition, H-MoN_5_@PtN_4_/C could degrade overexpressed glutathione (GSH) via redox reactions, thus effectively avoiding ROS depletion. Therefore, H-MoN_5_@PtN_4_/C can overcome the limitations of complex TME and kill tumor cells with high selectivity and efficiency [95]. Wang et al. successfully synthesized four noble metal-porphyrins (MxP, with *x* standing for Pt, Pd, Ru, and Ir) to mimic the active site of HRP through metal-N atom coordination anchored at the porphyrin center. Among them, MIrP provides the primary coordination bond through the N atom of the porphyrin structure, and the axially introduced O or Cl atoms enhance the metal-carrier binding energy through strong electronegativity and inhibit the migration of Ir atoms. The researchers found that the catalytic activity of MIrP exhibited much higher than that of the other MxPs and the classical MFeP, and that its optimal catalytic temperature and pH range were superior to that of HRP. In the meantime, the MIrP and MFeP were stored at room temperature for 0–16 weeks, and the absorption characteristics and catalytic performance of the materials at different storage times were examined. The results showed that MIrP with different storage times exhibited a stable absorption peak at 420 nm [96].

**Fig. 9:**
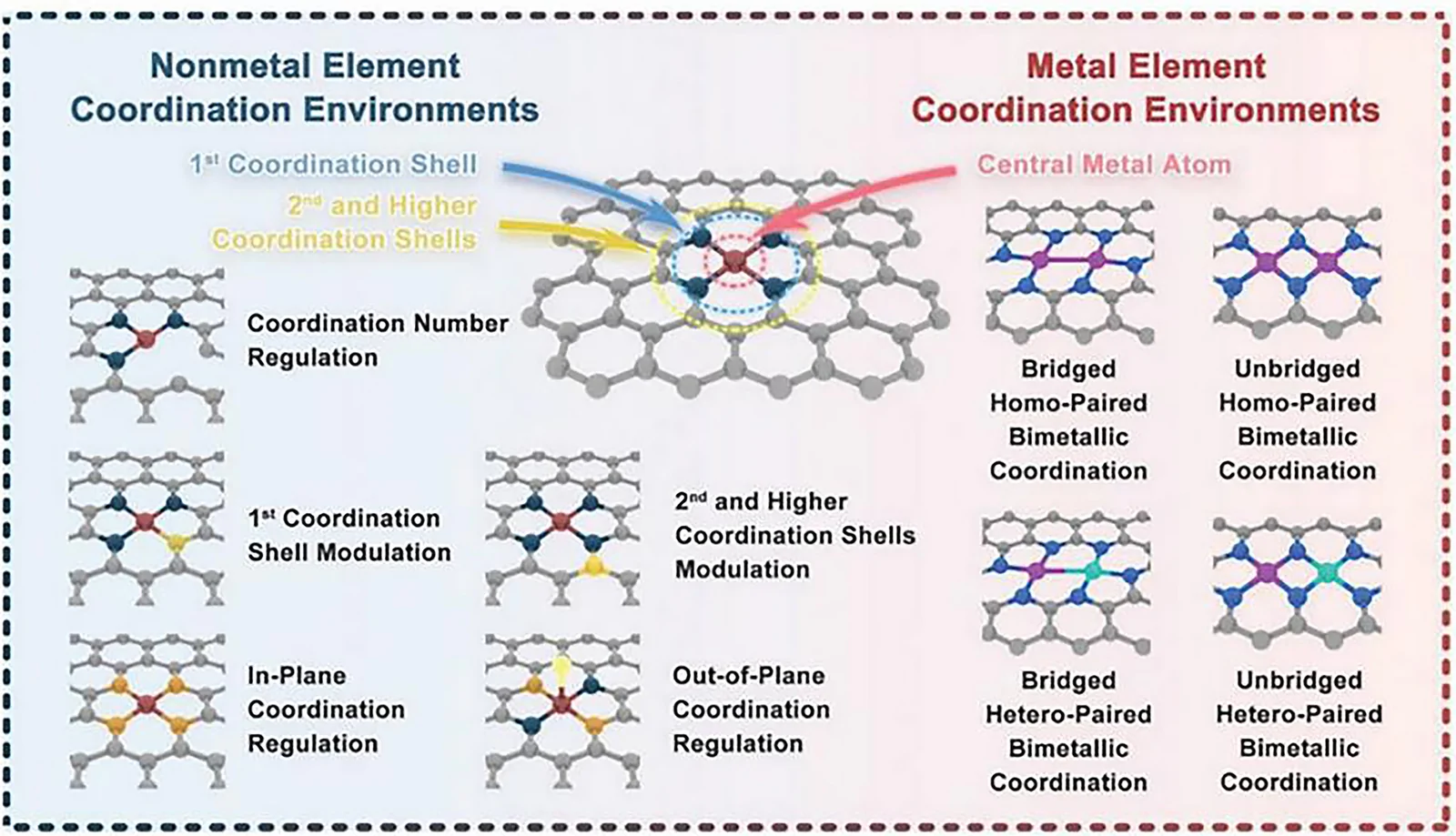
Altering various coordination environment strategies in carbon-based matrix. Reproduced with permission [79, 106]. Copyright 2023, Wiley

On the other hand, heteroatoms with different atomic radii and electronegativities (e.g., B, O, S, P, and F) can modulate the electronic structure of the central metal atoms in the second coordination shell layer through long-range electronic interactions (Fig. 9) [79, 97]. Heteroatom doping can break the planar symmetry of electron density in metal–nitrogen–carbon (M–N–C) and precisely regulate the electron distribution in the microenvironment of the active centers, thus significantly enhancing the activity [98]. For example, Li et al. found that phosphorus (P) atom doping can optimize the electronic structure of FeN_4_ sites. The distance effect of P to Fe enhances the activation of O_2_ by modulating the valence electrons and spin magnetic moments of Fe. Although the strong adsorption of ·OH intermediates at the P site may reduce the activity, the adsorption energy and reaction kinetics can be balanced to enhance the overall stability by rationally designing the doping distance, and the P-doped catalysts were observed to exhibit higher structural stability in acidic environments, lower solvation potentials, and prolonged electrochemical lifetimes [99]. Ji et al. engineered the FeN_3_P-SAzyme, which controlled the electronic structure of the single-atom iron active center by precise coordination of phosphorus and nitrogen, and exhibited POD catalytic activity and kinetics comparable to those of the natural enzyme [83]. Chen et al. doped Fe–N–C catalysts (Fe_1_-NC) with sulfur (S) atoms and found that the introduction of S atoms altered the spin state of Fe to form a low-spin state Fe^3+^ active site (C-FeN_4_-S configuration), in which S has a lower electronegativity, optimized the electronic structure of Fe, and facilitated the detachment of ·OH intermediates, thereby significantly enhancing the oxygen reduction reaction (ORR) activity. In addition, the participation of S atoms in the secondary coordination sphere enhances the metal-carrier interaction and inhibits the migratory agglomeration of Fe atoms [100]. Fe-N_3_S_1_ synthesized by Jiao et al. with asymmetric coordination showed higher POD-like activity than Fe-N_4_ characterized FeNC [101]. Sun et al. constructed PEG@P@Ce-N/S-C for multimodal assay of butyrylcholinesterase activity. In this case, the catalytic center of Ce SAzymes was constructed in the configuration of Ce-N_4_S_2_-C, and the S atom was symmetrically locked in the second ligand shell layer, which modulated the electronic state of the central Ce atom and enhanced its affinity for substrates. Additionally, in situ polymerization of dopamine on the ZIF surface remodels the carbon carrier. The polyphenol oxidase activity of the cerium source enables the in situ polymerization of dopamine on the surface of the precursor and avoids the detrimental effect of conventional alkaline polymerization conditions on the structure of the precursor. It was demonstrated that the Ce SAzymes had significant advantages in terms of catalytic kinetic parameters [102]. Tang et al. synthesized Cu–Cl MOFs with excellent enzyme-like activity by precisely regulating the coordination of halogen atoms, which could decompose ROS into H_2_O and O_2_, and meanwhile, exhibited remarkable antioxidant and anti-apoptotic functions in human corneal epithelial (HCE) cells by regulating Nrf2 and JNK or p38 MAPK in vitro. Stability experiments revealed that the catalytic activity of Cu–Cl MOFs hardly decreased after 90 days of storage [103].

In addition, the polyatomic coordination of N, P, and S can form stable structures [104]. For example, Chen et al. enhanced the metal-carrier interactions by introducing three kinds of coordination atoms, N, P, and S, to form the Pt_1_–N_3_PS active site, in which the N atom acts as the primary coordination atom to provide stable Pt–N bonding and inhibit atomic migration; the P atom regulates the d-band center position of Pt through the electron-donating effect to optimize the intermediate adsorption energy; and the strong electronegativity of the S atom further stabilizes the coordination structure and enhances the resistance to high-temperature oxidation, and the strong covalent bond formed by multi-atom coordination effectively inhibits the agglomeration of Pt atoms during high-temperature (900 °C) calcination. Experiments showed that the POD activity of Pt_1_–N_3_PS nanoenzymes reached 1 million/M·s, which was more than 100 times that of the traditional Pt nanoparticle catalysts, and there was no obvious decay of the activity after 30 h of continuous catalysis [105].

#### Bimetallic Synergistic Strategy

Stability enhancement by bimetallic (Fe/Co, Mn/Cu, etc.) synergy. For example, the oriented synthesis of Mo/Zn bimetallic monoatoms was achieved by carbonization of poly(vinyl alcohol) (PVA) aerogel as a three-dimensional substrate after infiltration with supramolecular complexes/heteropolyacid precursors, in which Mo and Zn monoatoms optimize each other’s d-band centers through electron transfer to reduce the diffusion barriers of the ·OH radicals in the catalytic reaction and enhance the POD activity, and the porous carbon carriers formed after carbonization of PVA aerogels inhibit migratory agglomeration by anchoring metal atoms through oxygen-containing groups. It was observed that the metal atoms of Mo/Zn bimetallic SAzymes remained dispersed after one year of immersion in deionized water, and the catalytic activity was not significantly attenuated [107]. Ning et al. designed a cascade diatomic nanoenzyme (FeCu-DA) with iron and copper sites for cancer immunotherapy by synergistically enhancing the activities of POD and SOD. The iron and copper sites in FeCu-DA improved the stability and catalytic performance of the nanoenzyme by synergizing the iron sites with POD activity and the copper sites with SOD activity, which were synergistically able to generate ROS more efficiently, enhance the effect of ICD, and improve the efficiency of cancer immunotherapy [108]. Ma et al. constructed PtNPs-Fe/NC, a bimetallic catalyst with a synergistic multiscale catalytic site with low content of Pt nanoparticles modifying Fe–N–C, for oxygen reduction reaction (ORR). In this case, there was a significant directional electron transfer path between the Pt NPs and Fe metal sites, and this directional electron transfer promoted a synergistic electronic effect between the metal sites, which in turn enabled the effective regulation of the electron density around the Fe sites. This synergistic effect improved the stability and catalytic performance of the catalyst, which led to its excellent catalytic activity and stability in ORR [109].

#### Defect Engineering Strategy

By constructing defects on the carrier surface, the presence of these defects alters the surrounding electronic structure and coordination environment, resulting in the appearance of a number of coordination-unsaturated defect sites on the carrier [110], which can be used to adsorb mononuclear metal precursors and anchor metal single atoms, thus realizing the synthesis of SAzymes. Zhang et al. rationally designed an iron-based SAzyme (Fe-SAzyme) by edge site engineering, which centrally exposed the edge defects anchored in the hierarchical mesoporous structure Fe-N_4_ atomic site defects induced a significant charge transfer from Fe atoms to the carbon matrix, which led to a more activated central Fe, thus enhancing the H_2_O_2_ interaction, weakening the O–O bond, and effectively catalyzing the decomposition of H_2_O_2_ into O_2_ and H_2_O with catalytic kinetic K M values superior to those of natural catalase and reported nanoenzymes. By performing CAT catalysis, Fe-SAzyme significantly scavenges ROS and attenuates oxidative stress, thereby eliminating pathological angiogenesis in an animal model of retinal vascular disease without affecting the repair of normal blood vessels [111]. Liu et al. synthesized a ligand-unsaturated Cu SAzyme loaded on sintered CeO_2_, and induced the transformation of inert Cu_1_O_4_ into ligand-unsaturated Cu_1_O_3_ sites by high-temperature baking, and this Cu_1_O_3_ active site with unsaturated ligand, as a new type of defective site, can greatly activate isolated Cu atoms and accelerate the dissociation of H_2_O_2_ to form ·OH, which results in a high POD activity of the SAzymes. The nanoenzymes have high POD activity, and at the same time, it is proved that the nanoenzymes have low cytotoxicity [112].

#### Atom Stripping-capture Strategy

The metal monoatoms in this strategy are derived from metal nanoparticles or bulk metal powders. In the process of synthesizing monoatomic sites, it is first necessary to create a suitable synthesis environment so that the metal atoms are gradually stripped from the metal nanoparticles or metal powders [113], and migrate to specific carriers, which are captured and anchored by the ligand sites on the carriers to form SAzymes. The key to the atom-stripping-capture strategy is to create suitable synthesis conditions that induce metal–metal bond breakage and allow the stripped metal atoms to form strong interactions with the new carriers [114], to anchor the metal single atoms and prevent their further migratory agglomeration. Chen et al. directly atomized platinum NPs into single atoms by reversing the calcination process to obtain high-performance SAzymes, in which the Pt atoms were gradually stripped from the surface of the Pt NPs driven by the high temperature, and then were captured by the anchoring sites of the N, P, and S co-doped carbon substrate to form thermally stabilized Pt single-atoms, and the Pt SAzyme with active Pt-N_3_PS sites and active Pt-N_4_ sites. The results showed that the prepared thermally stabilized Pt SAzyme (Pt_TS_-SAzyme) possessed significant POD activity and kinetics that far exceeded that of the PtNPs nanoenzymes [77].

### Surface Modification

**Fig. 10:**
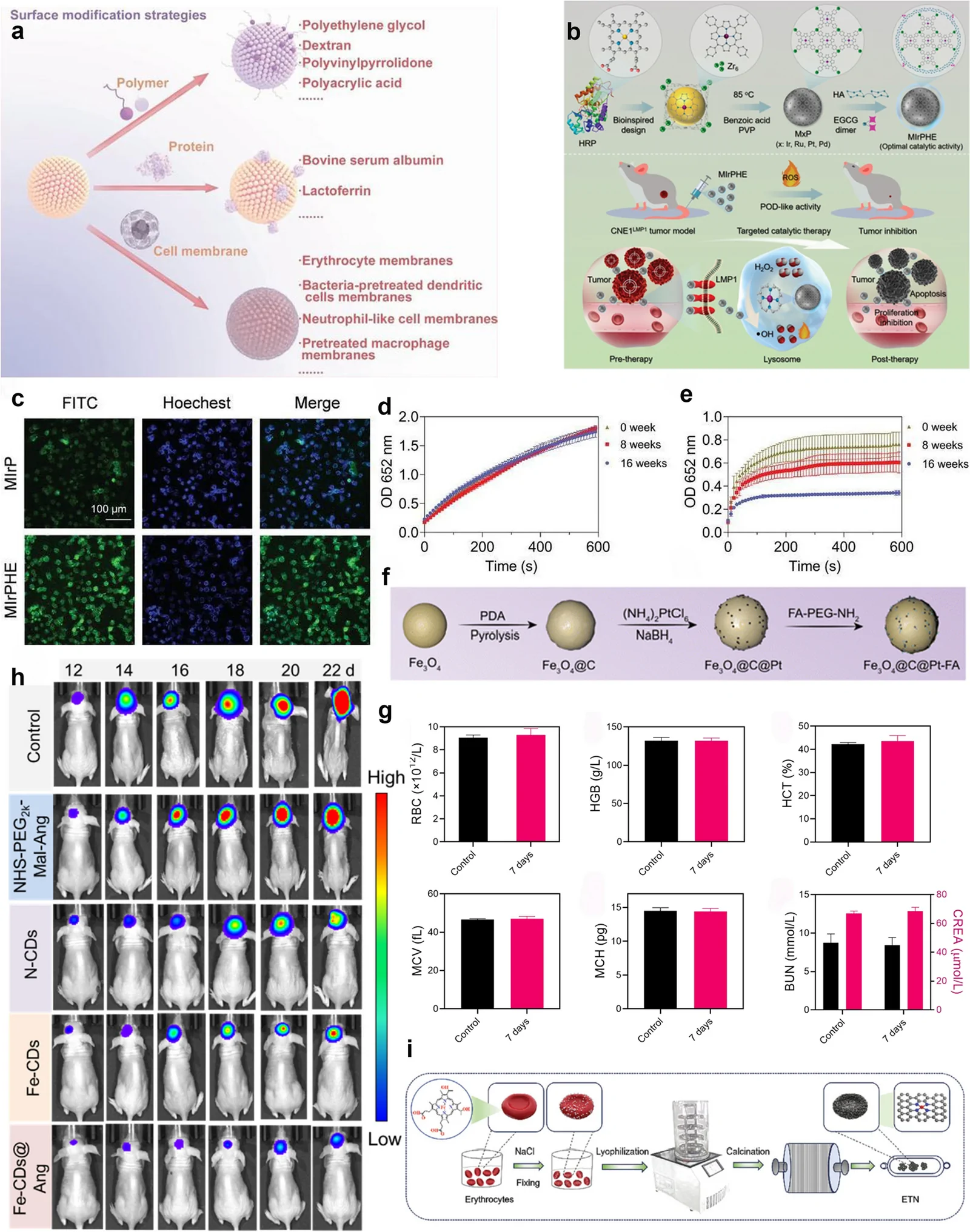
**a** Schematic illustration of surface modification strategies to improve the biocompatibility of nanozymes [115]. Copyright 2022, Wiley. **b** Schematic illustration of the synthesis process and therapeutic effect of MIrP [96]. Copyright 2024, Wiley. **c** Photographs and targeted effect of MIrPHE for CNE1^LMP1^ cells [96]. Copyright 2024, Wiley. Reaction-time curves of the TMB colorimetric reaction catalyzed by **d** MIrP and **e** MFeP stored at room temperature for 0–16 weeks (*n* = 3) [96]. Copyright 2024, Wiley. **f** vSchematic representation of the preparation process of the Fe_3_O_4_@C@Pt [116]. Copyright 2025, Royal Society of Chemistry. **g** Blood biochemical analysis of mice 1 week after injection of Fe_3_O_4_@C@Pt-FA [116]. Copyright 2025, Royal Society of Chemistry. **h** Tumor-bearing mice. Data are presented as mean SD (*n* = 6, one-way ANOVA and Tukey multiple comparisons tests, *p < 0.05, ***p < 0.001) [50]. Copyright 2022, Elsevier. **i** A schematic of synthesis process of ETN [119]. Copyright 2024, Wiley

Targeted molecular modifications—via aptamer, antibody, or peptide conjugation—enable precise, ligand-directed delivery of SAzymes while markedly curbing off-target accumulation. The surface modification strategies that have proven effective for boosting the biocompatibility and targeting specificity of nanozymes are readily adaptable to SAzymes (Fig. 10a) [115]. For example, Wang et al. designed a noble metal-porphyrin SAzyme (MIrPHE), by modifying the EBV-encoded LMP1 natural ligand on the surface, which specifically binds to the LMP1 receptor on the surface of the tumor cells (Fig. 10b), and significantly reduces the distribution of the nanoenzymes in normal tissues to achieve the precise targeting of EBV-associated nasopharyngeal carcinoma. In vivo experiments, the enrichment of MIrPHE in tumor sites was more than 3 times that of the unmodified nanoenzymes, without triggering obvious systemic toxicity, and stability experiments proved that MIrPHE has good stability (Fig. 10c–e) [96]. Folate receptor (FR) is highly expressed on the surface of a variety of tumor cells (e.g., ovarian cancer, breast cancer). Li et al. covalently coupled a folate molecule to the surface of Fe–N–C SAzymes, which enabled active targeting of tumor cells through the specific binding of folate to FR and the release of ROS in the tumor microenvironment to kill cancer cells (Fig. 10f). Through near-infrared fluorescence imaging and small animal in vivo imaging, it was shown that the accumulation of folate-modified nanoenzymes in the tumor site was 3.2 times higher than that of the unmodified group, whereas the distribution in the liver and spleen was reduced by 40%, while the tumor volume inhibition rate was enhanced from 52% to 85% of the normal nanoenzymes without significant toxicity to normal tissues(Fig. 10h) [116]. Pir Muhammad et al. developed ultrasmall carbon dot-loaded iron SAzymes (Fe-CDs), which have the properties of several natural enzymes and are able to effectively cross the blood–brain barrier and selectively target glioblastomas through surface-modified peptides. Studies have shown that the cascade enzyme activity and cellular autophagy pathway of Fe-CDs can effectively inhibit tumor growth in a drug-resistant glioblastoma mouse model, demonstrating their potential for targeted therapeutic applications (Fig. 10g) [50]. Mechanochemical-assisted pyrolysis and post-synthesis protein engineering strategies were used to produce SAzymes with bioaffinity and cascade reactivity of the bioepidermal membrane, and the surface-modified lectin Cutter’s Protein A (ConA) could effectively localize the glycocalyx structure of the bioepidermal membrane and catalyze endogenous glucose to trigger a multiple cascade reaction with pH-adaptive properties, which could consume glucose and glutathione and generate ·OH radicals. BioSAzyme is biocompatible in vivo, as shown by its surface modification with the lectin ConA, which can effectively localize the glycocalyx structure of the bio-permembrane and catalyze the endogenous glucose to initiate multiple pH-adaptive cascade reactions to consume glucose and glutathione, and to generate ·OH radicals [117]. Wang et al. engineered Ag-doped zinc selenide (ZnSe) QDs with atomically dispersed superficial FeIII (FAQD) via a reverse cation exchange strategy, and a gelatinase (MMP)-cleavable assembled peptide (P1, CGGGKLVFFPLGVRG) linked with hydrophilic PEG (P1-PEG) was modified on FAQD (FAQD-1). At the tumor site, overexpressed MMP cleaves the peptide (PLGVRG) in P1-PEG, removing PEG and allowing the self-assembling peptide (KLVFF) to promote the formation of nanoclusters through hydrogen bonding and hydrophobic interactions [118].

In addition, biocompatibility can be enhanced and metabolism can be promoted by using natural materials (e.g., ferritin, cellulose) as templates. For example, erythrocyte-templated nanozyme (ETN) is erythrocyte-based SAzymes, which are prepared by cell immobilization, salting out, and pyrolytic carbonization using hemoglobin from erythrocytes as the iron source (Fig. 10i). ETN have POD activity and can catalyze the production of ·OH radicals, and its catalytic activity can be further enhanced by near-infrared (NIR) light irradiation. In addition, the honeycomb structure of ETN can be used as a nanosponge to promote blood coagulation, accelerate hemostasis, and effectively kill methicillin-resistant Staphylococcus aureus (MRSA) to promote the healing of infected wounds, and its natural honeycomb structure facilitates degradation and reduces the long-term toxicity of ETN [119].

### Dynamic Responsive Design

**Fig. 11:**
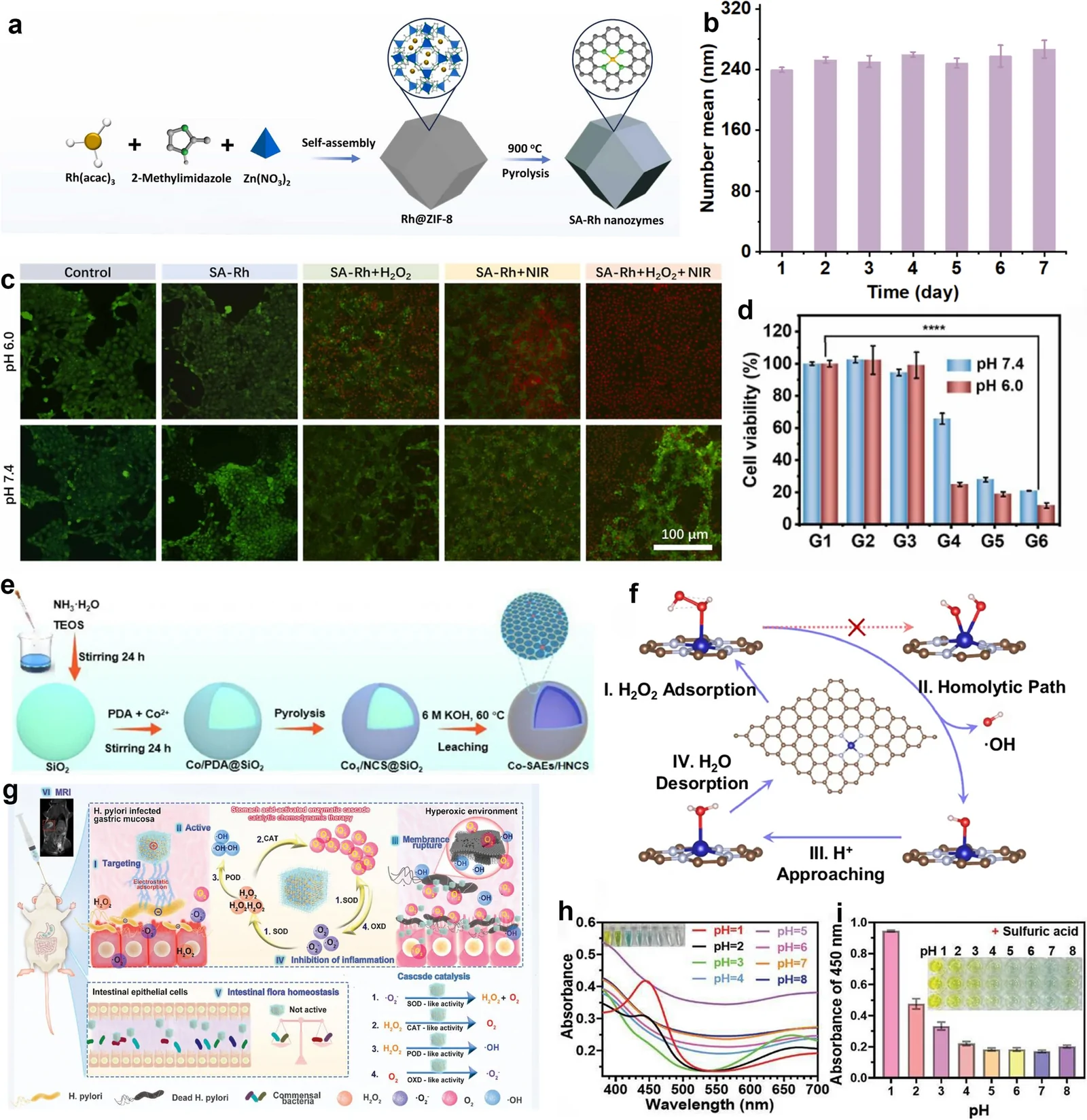
**a** Schematic illustration of the synthesis and anti-tumor therapy using the Rh-single-atom nanozyme [120]. Copyright 2024, ScienceDirect. **b** Change in hydrodynamic diameter of HCS-FeCu NEs at different days [120]. Copyright 2024, ScienceDirect. **c** Fluorescence images of 4T1 cells staining with calcein-AM (green) and PI (red) after different treatments and pH. Scale bar = 100 μm [120]. Copyright 2024, ScienceDirect. **d** Cell viability of 4T1 cells after different treatment with different pH. (G1: Control, G2: SA-Rh, G3: H_2_O_2_, G4: SA-Rh + H_2_O_2_, G5: SA-Rh + NIR, G6: SA-Rh + H_2_O_2_ + NIR [120]. Copyright 2024, ScienceDirect. **e** The schematic illustration of the synthesis of Co-SAEs/HNCS [90]. Copyright 2025, Springer Nature. f The proposed catalytic mechanism of ·OH caused by Co-SAEs/HNCS at pH 6.5 and 25 °C [90]. Copyright 2025, Springer Nature. **g** Schematic fabrication and application of the FPB-Co–Ch nanozyme composite [121]. Copyright 2024, Wiley. **h** Absorbance spectra and visual color changes of FPB-Co NPs + TMB in different pH solutions after 30 min incubation [121]. Copyright 2024, Wiley. **i** Absorbance spectra and visual color changes of FPB-Co NPs + TMB + H_2_O_2_ in different pH solutions after 30 min incubation [121]. Copyright 2024, Wiley

Environmentally responsive modulation involves engineering nanozymes whose activity can be switched on or amplified by pH, light, or enzymatic triggers under specific conditions. For example, Tao et al. constructed a pH-responsive SAzyme (SA-Rh) platform for synergistic oncology therapy by integrating Rh SAzymes and photothermal therapy (PTT) (Fig. 11a). SA-Rh SAzymes exhibit POD activity in tumor cells and are more efficiently catalyzed under acidic conditions, thus effectively exploiting the characteristics of the tumor microenvironment and enhancing their biocompatibility (Fig. 11b–d) [120]. Dai et al. embedded atomically dispersed Co-SAEs onto HNCS supports, named Co-SAE/HNCS (Fig. 11e), and prepared Co-SAEs/HNCS as an efficient photodynamic-photocatalytic-photothermal therapeutic agent to trigger the interactive ROS dynamic effect and thermodynamic effect by mutual fulfillment of multiple pathways in the TME, and the experimental results showed that, in the near-infrared irradiation, the photoelectron effect and photothermal effect simultaneously triggered the ROS burst and the local mild temperature increase under NIR irradiation. In addition, density-functional theory (DFT) calculations show that the highly atomically dispersed Co-N_4_ activity centers significantly enhance the excellent ROS dynamic activity of Co-SAEs/HNCS in the NIR-I region (Fig. 11f). The interactive ROS dynamic and thermodynamic effects effectively enhance biocompatibility [90]. Tong et al. designed and prepared a core–shell structure-based nanoenzymatic cascade oxygen production system (FPB-Co–Ch NPs) by modifying Co_3_O_4_ NPs on the surface of novel ferrocene-doped Prussian blue analogs (FPB NPs) as the core, and using chitosan, which has good biocompatibility and bioadhesion, as the coating, under acidic conditions (Fig. 11g). The nanoenzymatic system FPB-Co–Ch NPs could exert four enzymatic activities, which generated oxygen by mimicking SOD and CAT activities, produced hydroxyl radicals by POD-like activity, and promoted the cascade reaction by mimicking OXD activity. Under in vitro conditions, the FPB-Co–Ch NPs system showed good killing efficacy against both sensitive and drug-resistant H.pylori, and this bactericidal efficacy had a significant pH-dependence, and the results of the in vitro experiments showed that the FPB-Co–Ch NPs also exhibited some anti-inflammatory and tissue repair functions in vivo, had no significant effect on the intestinal flora, and had good biocompatibility (Fig. 11h, i) [121]. Yao et al. developed CaCO_3_-based pH-programmed responsive iron single-atom nanoparticles (SAF NPs) that achieve pH-programmed release of DOX at tumor sites, improving targeting and anticancer activity [122].

## Conclusion and Perspectives

As new generation of nanozymes, SAzymes have recently achieved breakthroughs in catalytic science and biomedicine. In disease therapy, SAzymes selectively eradicate tumor cells via ROS generation, combat drug-resistant bacteria, or promote infected wound healing. In biosensing, they enable ultrasensitive biomarker detection for real-time disease monitoring. This review summarized the stability issues of SAzymes, such as metal atom aggregation and loss of active sites, ligand bond cleavage at high temperatures, insufficient environmental tolerance, biosafety risks, and limited long-term catalytic stability, and proposes potential countermeasures, including synthesis process optimization (space-limited strategy, coordination site design, bimetallic synergistic strategy, defect engineering strategy, atom stripping-capture), surface modification, and dynamic responsive design. Yet each approach has merits and drawbacks: coordination site design and defect engineering strengthen metal–support bonding, but operate within narrow defect-concentration windows and are prone to configurational drift; space-limited strategy can fully suppress aggregation at the cost of diffusion limitations and scale-up expense; surface modification improves biocompatibility but may mask active sites or detach in physiological milieus; dynamic responsive design is sustainable yet constrained by the degradation of repair molecules and system complexity.

It must be emphasized that structure dictates both activity and stability: the local coordination environment-M-N_*x*_, M-C_*x*_, or M-N_*x*_Cl_γ_-tunes the electronic structure of the single-atom center, governing substrate adsorption, activation, and product release (e.g., CuN_3_ outperforms CuN_4_ in H_2_O_2_ affinity and kinetics), while the same motifs control thermodynamic and kinetic stability (Fe-N_4_ in defective graphene resists demetalation under oxidative stress; CuN_3_ retains geometry and activity after 500 Gy X-ray irradiation). From this “structure–activity–stability” perspective, clinical translation of SAzymes demands more than incremental stability gains; it requires a four-dimensional, synergistic roadmap:

Structure: move from empirical design to predictive modeling by constructing comprehensive structure–function databases that link atomic coordination, electronic structure, and catalytic performance.

Activity: break the activity–selectivity bottleneck through hierarchical cascade catalysis and microenvironment-responsive activation to ensure on-demand, site-specific catalysis.

Stability and Biocompatibility: engineer biodegradable scaffolds and surface modifications (e.g., cell-membrane cloaking, PEGylation) that evade the mononuclear phagocyte system, prolong circulation half-life, and eliminate metal-ion accumulation toxicity.

Manufacturing and Safety: develop GMP-compatible synthetic routes (e.g., low-temperature MOCVD), rigorously assess ADME profiles and immunogenicity, and validate efficacy/toxicity in microfluidic tumor-on-chip and organoid models.

To advance from bench to bedside, SAzymes must transcend the simplistic notion that “stable equals successful” and embrace this four-dimensional roadmap: structure-predictable, activity-tunable, biocompatible, and scalable. Only by integrating these four axes can we transform SAzymes from “star materials” of the laboratory into precise clinical tools for medicine.
